# LMO3 reprograms visceral adipocyte metabolism during obesity

**DOI:** 10.1007/s00109-021-02089-9

**Published:** 2021-05-20

**Authors:** Gabriel Wagner, Anna Fenzl, Josefine Lindroos-Christensen, Elisa Einwallner, Julia Husa, Nadine Witzeneder, Sabine Rauscher, Marion Gröger, Sophia Derdak, Thomas Mohr, Hedwig Sutterlüty, Florian Klinglmüller, Silviya Wolkerstorfer, Martina Fondi, Gregor Hoermann, Lei Cao, Oswald Wagner, Florian W. Kiefer, Harald Esterbauer, Martin Bilban

**Affiliations:** 1 Department of Laboratory Medicine, Medical University of Vienna, 1090 Vienna, Austria; 2 Clinical Division of Endocrinology and Metabolism, Department of Medicine III, Medical University of Vienna, 1090 Vienna, Austria; 3 Present Address: Novo Nordisk, Maaloev, Denmark; 4 Core Facilities, Medical University of Vienna, 1090 Vienna, Austria; 5 Institute of Cancer Research, Department of Medicine I, Comprehensive Cancer Center, Medical University of Vienna, 1090 Vienna, Austria; 6 Center for Medical Statistics, Informatics, and Intelligent Systems, Medical University of Vienna, 1090 Vienna, Austria; 7 Present Address: Austrian Medicines & Medical Devices Agency, 1200 Vienna, Austria; 8 University of Applied Sciences, FH Campus Wien, 1100 Vienna, Austria; 9 Present Address: Institute of Cardiovascular Prevention, Ludwig-Maximilians-University, 80336 Munich, Germany; 10 Central Institute of Medical and Chemical Laboratory Diagnostics, University Hospital Innsbruck, 6020 Innsbruck, Austria; 11 Department of Cancer Biology and Genetics, The Ohio State University, Columbus, OH 43210 USA

**Keywords:** LMO3, Obesity, Visceral adipose tissue

## Abstract

**Abstract:**

Obesity and body fat distribution are important risk factors for the development of type 2 diabetes and metabolic syndrome. Evidence has accumulated that this risk is related to intrinsic differences in behavior of adipocytes in different fat depots. We recently identified LIM domain only 3 (LMO3) in human mature visceral adipocytes; however, its function in these cells is currently unknown. The aim of this study was to determine the potential involvement of LMO3-dependent pathways in the modulation of key functions of mature adipocytes during obesity. Based on a recently engineered hybrid rAAV serotype Rec2 shown to efficiently transduce both brown adipose tissue (BAT) and white adipose tissue (WAT), we delivered YFP or *Lmo3* to epididymal WAT (eWAT) of C57Bl6/J mice on a high-fat diet (HFD). The effects of eWAT transduction on metabolic parameters were evaluated 10 weeks later. To further define the role of LMO3 in insulin-stimulated glucose uptake, insulin signaling, adipocyte bioenergetics, as well as endocrine function, experiments were conducted in 3T3-L1 adipocytes and newly differentiated human primary mature adipocytes, engineered for transient gain or loss of LMO3 expression, respectively. AAV transduction of eWAT results in strong and stable *Lmo3* expression specifically in the adipocyte fraction over a course of 10 weeks with HFD feeding. LMO3 expression in eWAT significantly improved insulin sensitivity and healthy visceral adipose tissue expansion in diet-induced obesity, paralleled by increased serum adiponectin. In vitro, LMO3 expression in 3T3-L1 adipocytes increased PPARγ transcriptional activity, insulin-stimulated GLUT4 translocation and glucose uptake, as well as mitochondrial oxidative capacity in addition to fatty acid oxidation. Mechanistically, LMO3 induced the PPARγ coregulator Ncoa1, which was required for LMO3 to enhance glucose uptake and mitochondrial oxidative gene expression. In human mature adipocytes, LMO3 overexpression promoted, while silencing of LMO3 suppressed mitochondrial oxidative capacity. LMO3 expression in visceral adipose tissue regulates multiple genes that preserve adipose tissue functionality during obesity, such as glucose metabolism, insulin sensitivity, mitochondrial function, and adiponectin secretion. Together with increased PPARγ activity and Ncoa1 expression, these gene expression changes promote insulin-induced GLUT4 translocation, glucose uptake in addition to increased mitochondrial oxidative capacity, limiting HFD-induced adipose dysfunction. These data add LMO3 as a novel regulator improving visceral adipose tissue function during obesity.

**Key messages:**

- LMO3 increases beneficial visceral adipose tissue expansion and insulin sensitivity in vivo.
- LMO3 increases glucose uptake and oxidative mitochondrial activity in adipocytes.
- LMO3 increases nuclear coactivator 1 (Ncoa1).
- LMO3-enhanced glucose uptake and mitochondrial gene expression requires Ncoa1.

**Supplementary Information:**

The online version contains supplementary material available at 10.1007/s00109-021-02089-9.

## Introduction

Accumulation of visceral adipose tissue (VAT) correlates with metabolic abnormalities, whereas increased amounts of subcutaneous fat is thought to have neutral or even protective metabolic effects. To some extent, this correlates with depot-specific differences of mature adipocyte functions, including glucose homeostasis, insulin sensitivity, rate of lipolysis, and endocrine activity [1, 2].

We have previously identified the GC-dependent gene LIM domain only 3 (LMO3) as being selectively upregulated in a depot-specific manner in human obese visceral adipose tissue, localizing primarily in the adipocyte fraction [3, 4]. We showed that LMO3 is a new key player in the development of human adipose tissue, acting as a new partner in GC-dependent signaling to modulate the key adipogenic master switch PPARγ in human, but not mouse, visceral adipose progenitors. LMO3 expression is undetectable throughout differentiation of murine primary preadipocytes and remains undetectable in WAT of a genetic obesity mouse model (*db*/*db*) and HFD-challenged mice [3]. Remarkably, when Lmo3 was overexpressed in 3T3-L1 preadipocytes, it exerted the phenotype observed in human adipose stromal cells (hASCs), i.e., enhanced adipogenesis, implying that murine cells can utilize Lmo3, but that due to lack of conservation in the glucocorticoid response element 1 site, it is not inducible. While PPARγ is the master regulator of adipocyte development, proper PPARγ activity in the mature adipocyte is of equal importance considering that adipocytes only survive for a few days after selective ablation of PPARγ in mature adipocytes of mice [5–7]. PPARγ regulates metabolic homeostasis through direct regulation of genes involved in lipid metabolism, glucose homeostasis, as well as the expression of adipose secreted factors that act as transducers for PPARγ, including adiponectin [7]. In addition to posttranslational modifications, PPARγ activity is differentially regulated also by its association with various coregulators, including the p160/nuclear coactivator 1–3 family (also called steroid receptor coactivators SRC-1–3) [8]. Coregulator recruitment to PPARγ results in major differences in PPARγ transactivation and in alterations in glucose homeostasis, insulin sensitivity, and mitochondrial oxidative activity [9–12]. Signaling events and networks can be coordinated by adaptor proteins, which facilitate the proper localization of effector molecules, transcription factors, and kinases [13–15]. Adaptor proteins, such as those of the highly conserved LMO protein family, remain poorly understood compared with other classes of signaling molecules, especially in the context of metabolism.

Despite clear evidence that LMO3 is required for adipogenesis per se (recently confirmed by others [16]), the functions or targets of LMO3 in mature adipocytes are unknown. Genes induced in late adipogenesis including *LMO3* are expected to be involved in WAT functionality, as they likely fine-tune certain (depot-specific) aspects of adipocyte function [17]. Ways to genetically manipulate adipose tissue in the adult are fundamental to gain insight into gene functions within fat tissue. Genetic manipulation of fat has primarily relied on the use of transgenic mice. A main limitation of these models, however, is that transgene expression affects embryo development in an undesirable manner [18–20]. In contrast to adenoviral (AV) vectors, adeno-associated viral (AAV) vectors have been widely used for experimental applications in vivo because of their low immunogenicity and their ability to transduce post-mitotic tissue, enabling long-lasting gene expression [21, 22]. Here, we took advantage of a recently engineered AAV serotype Rec2 that preferentially targets adipose tissue, which has been used for metabolic and pathophysiology studies [22–24]. Here, we developed an AAV-based, visceral adipose tissue specific “*Lmo3* knock-in” mouse model to investigate the potential involvement of LMO3 in the modulation of key (visceral) adipocyte functions during obesity. Due to the depot-specific expression of LMO3 in VAT [3] and the clinical relevance of this adipose depot, we here focused on potential effects of Lmo3 specifically on visceral adipocytes.

## Results

### LMO3 rewires the metabolic transcriptional program in mature adipocytes

**Fig. 1:**
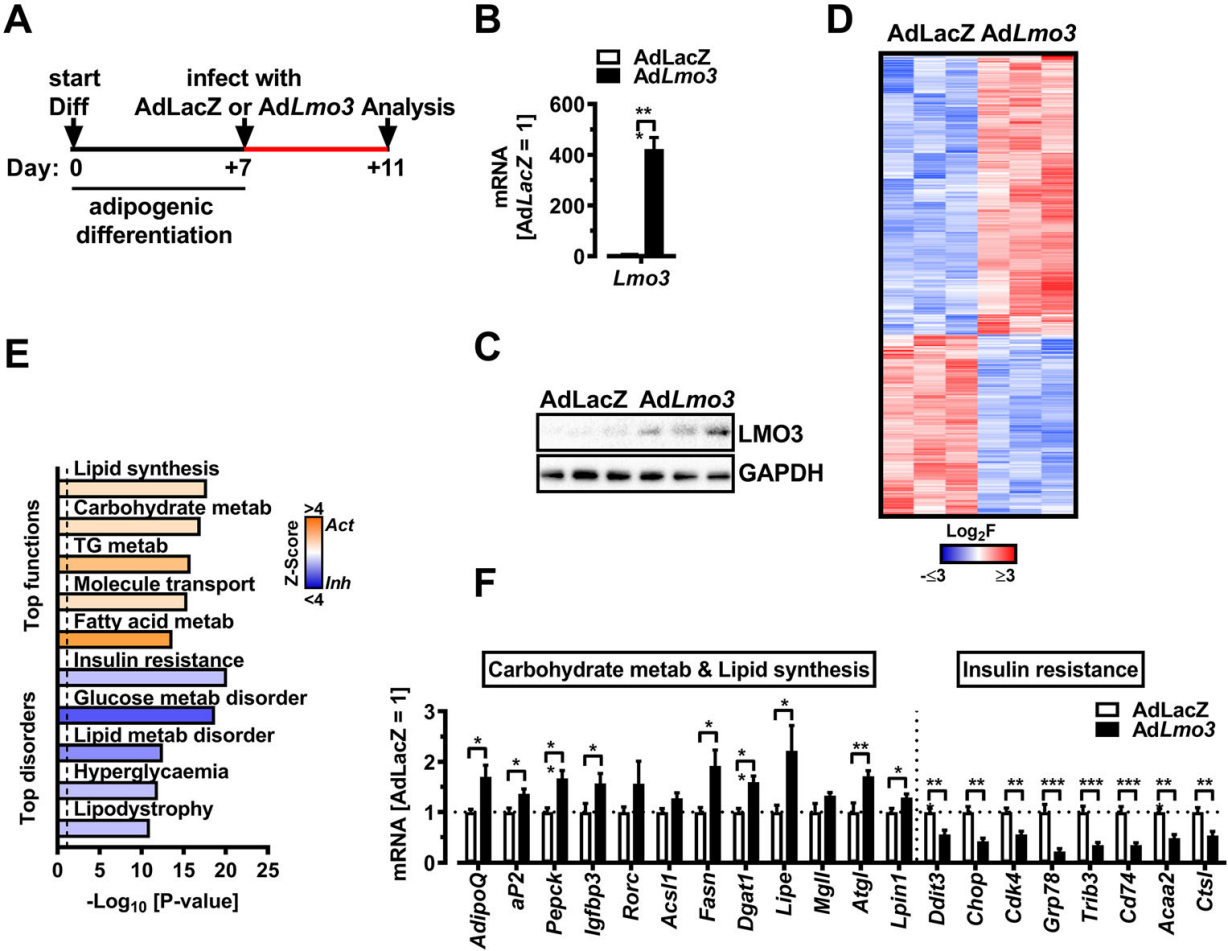
LMO3 rewires the metabolic transcriptional program in mature adipocytes. **A** Experimental design for analysis of LMO3 effects in mature 3T3-L1 adipocytes. **B** *Lmo3* mRNA expression in mature 3T3-L1 adipocytes 3 days after infection with a control (AdLacZ) or *Lmo3* containing (Ad*Lmo3*) adenovirus (n = 4). **C** LMO3 protein expression in mature 3T3-L1 adipocytes 3 days after infection with a control (AdLacZ) or *Lmo3* containing (Ad*Lmo3*) adenovirus (n = 3). GAPDH demonstrates equal protein loading. **D** Heatmap of 1093 genes differentially regulated between Ad*Lmo3*- and Ad*LacZ*-transduced 3T3-L1 adipocytes identified by RNA-Seq. Red and blue colors correspond to genes with statistically significant up- and downregulation, respectively (adjusted p-value < 0.05). **E** Ingenuity Pathway Analysis (IPA)-predicted molecular functions and disorders showing activation Z-scores (bars). Functions/disorders with an overlap p < 0.05 and Z-score −2 < or > 2 by IPA (see “Methods” for description) were predicted to be inhibited or activated. Dotted line indicates p < 0.05. **F** qRT-PCR analysis of selected genes from the Ingenuity Pathway Analysis (shown in G), (n = 5–7). IPA-predicted upstream regulators (center, colored by Z-score) and target genes (outer circle, colored by Fold Change) in Ad*Lmo3*- versus AdLacZ-transduced 3T3-L1 adipocytes

To begin to understand the effects of LMO3 on mature adipocyte function, we adenovirally overexpressed *Lmo3* in de-novo differentiated 3T3-L1 adipocytes. Adenoviral infection of de-novo differentiated 3T3-L1 cells on day 7 of differentiation resulted in efficient *Lmo3* mRNA and protein expression examined 48 h later (Fig. 1A–C). RNA-Seq analysis identified 1093 genes significantly regulated by LMO3 in 3T3-L1 cells, with 663 and 430 genes being induced or repressed, respectively (Table S1 and Fig. 1D). Ingenuity Pathway Analysis (IPA) software classified these genes into molecular functions implicated in lipid and carbohydrate metabolism/transport being activated (positive Z-score), while molecular features of insulin resistance, dyslipidemia, and hyperglycemia were all suppressed by LMO3 (negative Z-score), respectively (Fig. 1E), and further verified by Q-PCR (Fig. 1F).

### LMO3 regulates insulin-induced glucose uptake and GLUT4 translocation

**Fig. 2:**
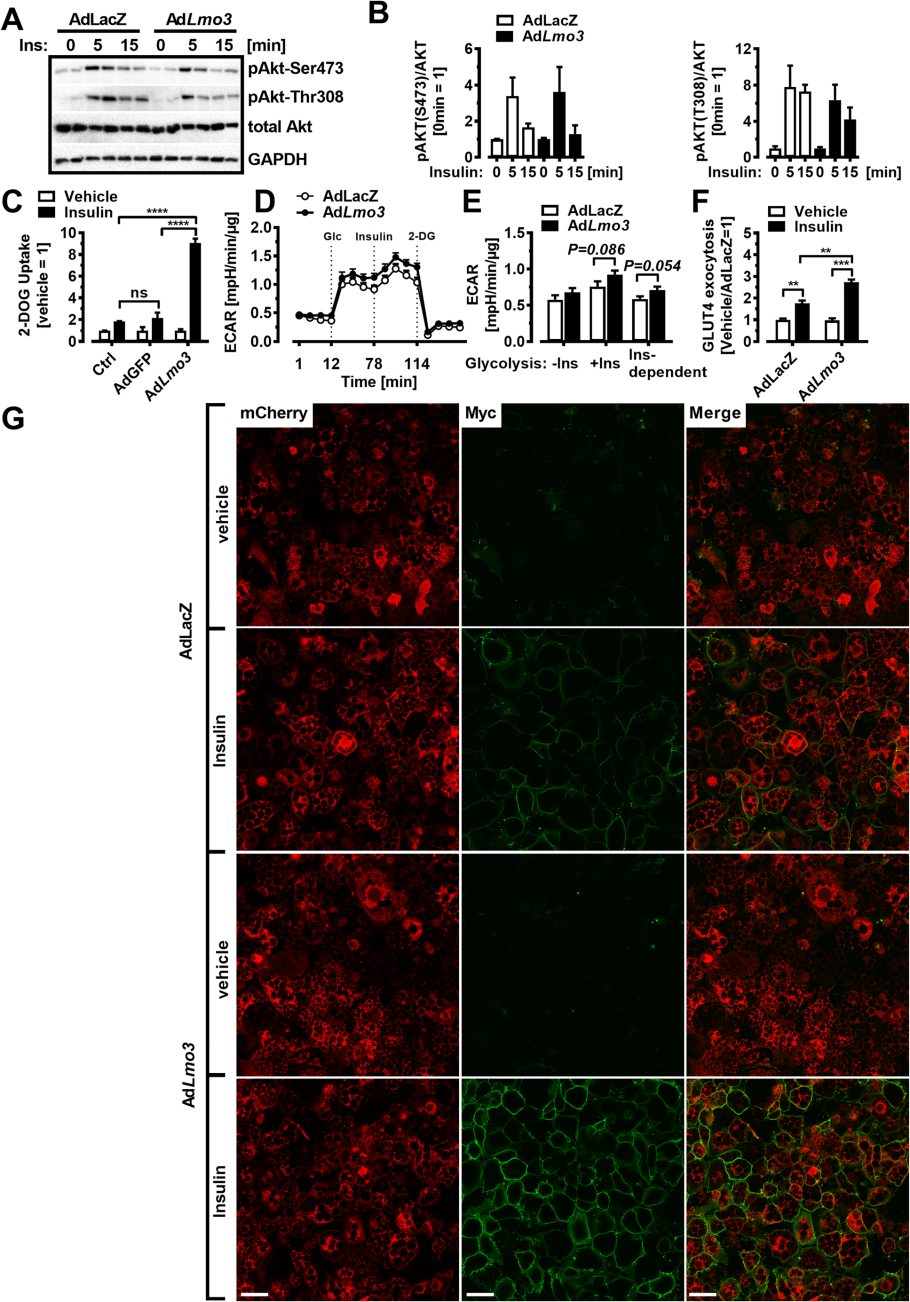
LMO3 augments insulin-induced glucose uptake and GLUT4 translocation. **A** Western blotting for phosphorylated and total AKT in AdLacZ- or Ad*Lmo3*-transduced mature 3T3-L1 adipocytes treated with insulin for the indicated times. GAPDH demonstrates equal protein loading. **B** Densitometry of AKT phosphorylation as shown in (**A**). **C** 2-deoxyglucose uptake in Ctrl, AdGFP, or Ad*Lmo3*-transduced mature 3T3-L1 adipocytes stimulated with or without insulin (100 nM). P-values were determined by 2-way ANOVA. **D** ECAR at baseline and after sequential treatment at the indicated time points with glucose (Glc, 5.5 mM), insulin (Ins, 100 nM), and 2-deoxyglucose (2-DG, 100 mM) in AdLacZ- or Ad*Lmo3*-transduced mature 3T3-L1 adipocytes. 2-DG was injected to inhibit glycolysis. **E** Insulin-dependent glycolysis in AdLacZ- or Ad*Lmo3*-transduced mature 3T3-L1 adipocytes, which was defined as the difference between insulin-induced glycolysis and basal glycolysis, normalized to total protein. See Figure S2B for detailed calculation. **F**, **G** Insulin-stimulated GLUT4 surface exposure in LMO3-overexpressing 3T3-L1 adipocytes. The cells received vehicle or insulin treatment for 15 min after 4-h serum starvation. The ratio of surface to total GLUT4 was quantified by detecting surface GLUT4 through anti-Myc fluorescence immunolabeling and total GLUT4 through mCherry fluorescence in non-permeabilized cells. Data in each group were normalized and expressed as a percentage of insulin-treated control cells. Scale bar: 50 μm. *p < 0.05, **p < 0.01, ***p < 0.001, ns, not significant

Next, we investigated if LMO3 targets insulin-induced AKT phosphorylation, which is central in controlling insulin effects on adipocytes. Treatment with insulin-induced phosphorylation at Ser473 as well as Thr308 of AKT to a similar magnitude in both, AdLacZ as well as Ad*Lmo3*, transduced 3T3-L1 adipocytes (Fig. 2A and B). Next, we investigated if insulin-induced glucose uptake in adipocytes is affected by LMO3. Indeed, insulin-stimulated glucose uptake was much greater in *Lmo3*-transduced 3T3-L1 adipocytes compared with non-infected control or AdGFP-transduced 3T3-L1 adipocytes (Fig. 2C). Of note, in the *absence* of insulin, basal, maximal, and reserve glycolytic capacity were similar, determined using the Seahorse technology (Figure S1A). However, insulin treatment resulted in higher extracellular acidification rates in *Lmo3*-transduced 3T3-L1 adipocytes presumably via increased (insulin-induced) glucose uptake (Fig. 2D and E; Figure S1B). This suggests that insulin sensitization by LMO3 uses an alternative pathway, e.g., its LIM domains could interfere with insulin-induced GLUT4 translocation by regulation of certain cytoskeletal components, as was shown previously for the related protein ENIGMA [25]. Since insulin-induced glucose uptake is primarily mediated by GLUT4 in adipocytes and *Glut4* mRNA levels were similar in Ad*LacZ*- and Ad*Lmo3*-transduced 3T3-L1 adipocytes (Figure S1C), we wanted to investigate if LMO3 alters GLUT4 translocation from the cytosol to the plasma membrane. We generated a 3T3-L1 cell line stably expressing a Myc-GLUT4-mCherry fusion protein in which the Myc epitope was inserted in the first exofacial loop of GLUT4 N terminus, and mCherry fused at the C terminus, thus allowing detection of GLUT4 on plasma membrane insertion (by Myc in non-permeabilized condition) and of total GLUT4 content (by mCherry) [26]. In control cells (AdLacZ), insulin induced GLUT4 presence at the cell surface; however, overexpression of LMO3 further increased the GLUT4 presence in insulin-treated cells to higher levels (Fig. 2F and G). These results indicate that LMO3 enhances insulin-stimulated glucose uptake by increasing GLUT4 translocation to the plasma membrane in adipocytes.

### *Lmo3* knock in via AAV-mediated transduction of eWAT

LMO3 is highly upregulated in human visceral AT; therefore, we generated an in vivo *Lmo3* knock-in mouse model specifically in epididymal WAT (eWAT) to delineate the function of LMO3 in mature adipocytes in murine visceral AT. Among several potential AAV serotypes, we applied a recombinant AAV serotype (rAAV) Rec2 featuring the highest transduction efficiency among seven serotypes to efficiently target WAT in mice [22–24].

### LMO3 expression in eWAT ameliorates obesity-induced metabolic dysfunction

**Fig. 3:**
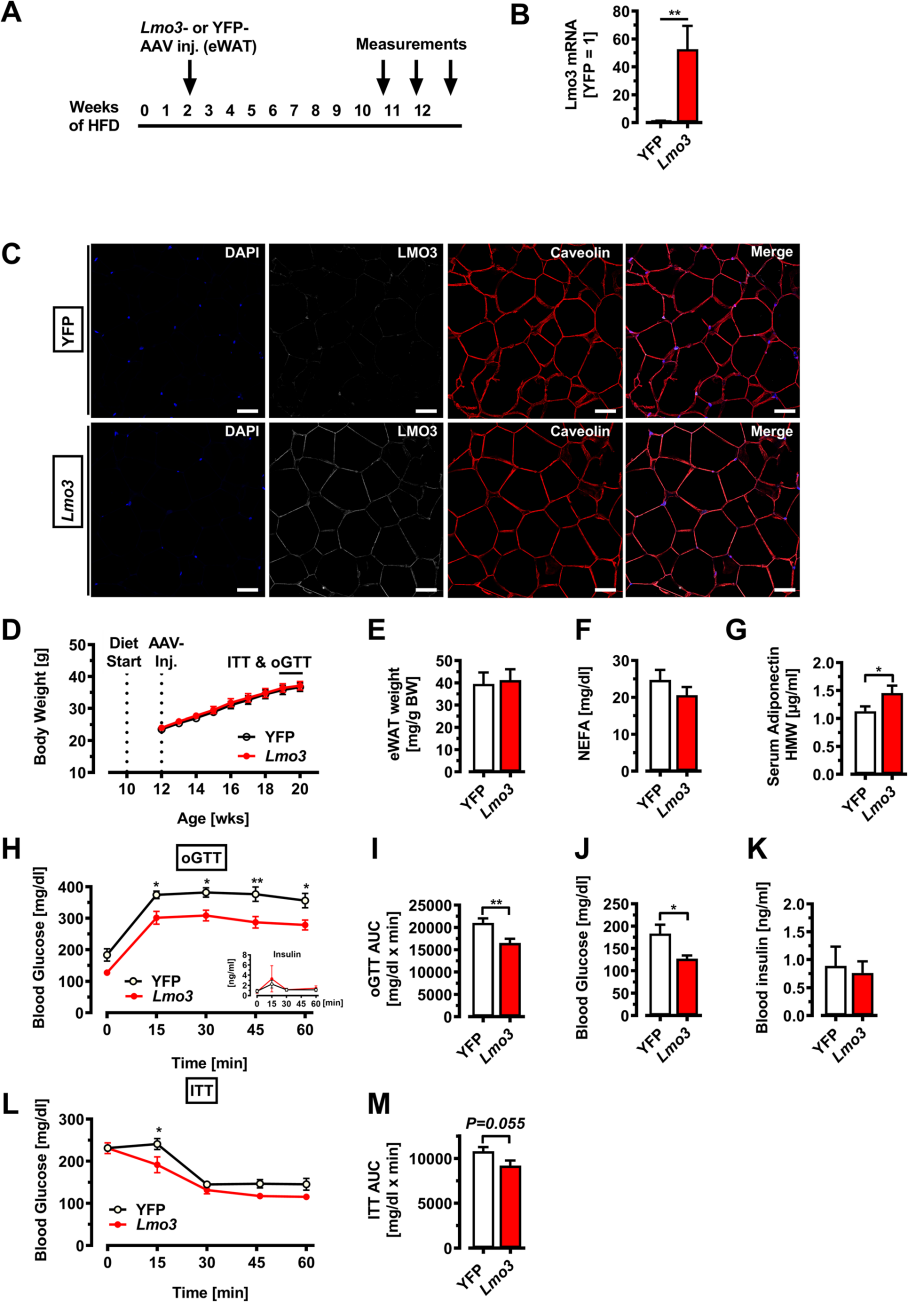
LMO3 increases glucose clearance and insulin sensitivity in eWAT during obesity. All mice were kept on HFD for 12 weeks and received rAAV-YFP or rAAV-*Lmo3* injections into eWAT at week 2 of HFD and were examined 10 weeks later. **A** Experimental scheme for murine studies. **B** Lmo3 mRNA expression in eWAT (n = 5/group). **C** Confocal immunofluorescence of eWAT. Scale bar = 50 μm. **D** Body weight gain over time (n = 8/group). **E** eWAT fat pad weight after 10 weeks of HFD (n = 6/group). **F** Serum free fatty acid levels (n = 10/group). **G** Serum adiponectin levels (n = 10/group). **H** Oral glucose tolerance test and corresponding blood glucose and insulin levels in obese mice (n = 6/group). **I** Area under the curve (AUC) for oGTT (n = 6/group). **J** Basal blood glucose levels in obese mice (n = 6/group). **K** Basal blood insulin levels in obese mice (n = 6/group). **L** Insulin tolerance test and corresponding blood glucose levels in obese mice (n = 6/group). **M** Area under the curve (AUC) for ITT (n = 6/group). *p < 0.05, **p < 0.01, ***p < 0.001, ns, not significant

To test whether LMO3 expression affects the function of mature adipocytes in eWAT during obesity, we injected rAAV-YFP or rAAV-*Lmo3* bilaterally, followed by 10 weeks on chow or HFD. Our goal was to focus on the role of LMO3 on mature adipocyte function rather than on adipogenesis, which we have described previously [3]; thus, we transduced eWAT not at the onset of HFD, but after an initial 2-week HFD period, when most obesogenic adipogenesis has already occurred [27, 28]. Metabolic effects as well as eWAT characteristics were evaluated 8–10 weeks later (Fig. 3A). This time point was chosen to detect adipocyte contributions to the development of metabolic disease, before the onset of macrophage infiltration [29], potentially obscuring LMO3 effects from adipocytes. After sacrificing the mice, *Lmo3* expression was evaluated in eWAT transduced with either rAAV-YFP or rAAV-*Lmo3*. qPCR demonstrated strong *Lmo3* expression in eWAT from rAAV-*Lmo3* animals (Fig. 3B), while LMO3 was neither detectable in eWAT from rAAV-YFP-transduced mice (data not shown) nor in iWAT, liver, or heart from rAAV-*Lmo3* as well as rAAV-YFP animals (Supplemental Figure S2B). Confocal immunofluorescence microscopy further demonstrates successful ectopic expression of LMO3 in the mature adipocyte fraction of rAAV-*Lmo3*, but not rAAV-YFP-transduced eWAT (Fig. 3C). Thus, rAAV transduction of eWAT can be used to efficiently and specifically target eWAT for gene transfer to assess the function of novel genes. Having verified strong LMO3 expression up to the end of the experiment (after transduction and 10 weeks of HFD) specifically in mature adipocytes, we next evaluated the metabolic effects of YFP- and *Lmo3*-transduced animals kept on HFD for 10 weeks. On normal chow, body weight, glucose tolerance, and insulin sensitivity of rAAV-*Lmo3*-transduced mice were not distinguishable from those of rAAV-YFP control mice (Supplemental Figure S2D and E). On HFD, rAAV-YFP- and rAAV-*Lmo3*-transduced mice gained weight to a similar extent and displayed similar fat pad weights (Fig. 3D and E). Circulating adiponectin levels were significantly elevated in rAAV-Lmo3 mice as compared to rAAV-YFP controls, while serum NEFAs tended to be reduced (Fig. 3F and G). Adiponectin is produced and secreted from fat and has been well established as an insulin-sensitizing adipokine [30]. While the rate of glucose clearance was similar in rAAV-Lmo3- and rAAV-YFP-transduced mice, basal glucose levels were significantly lower in mice that received rAAV-*Lmo3* injection (Fig. 3H–K). Insulin tolerance testing revealed the rAAV-*Lmo3*-transduced mice to be more sensitive to insulin than rAAV-YFP controls (Fig. 3L–M). Together, these data demonstrate that LMO3 stably overexpressed by AAV transduction in eWAT significantly improves glucose and tolerance, insulin sensitivity, and adiponectin secretion in mice fed a HFD.

### LMO3 targets PPARγ activity in eWAT during obesity

**Fig. 4:**
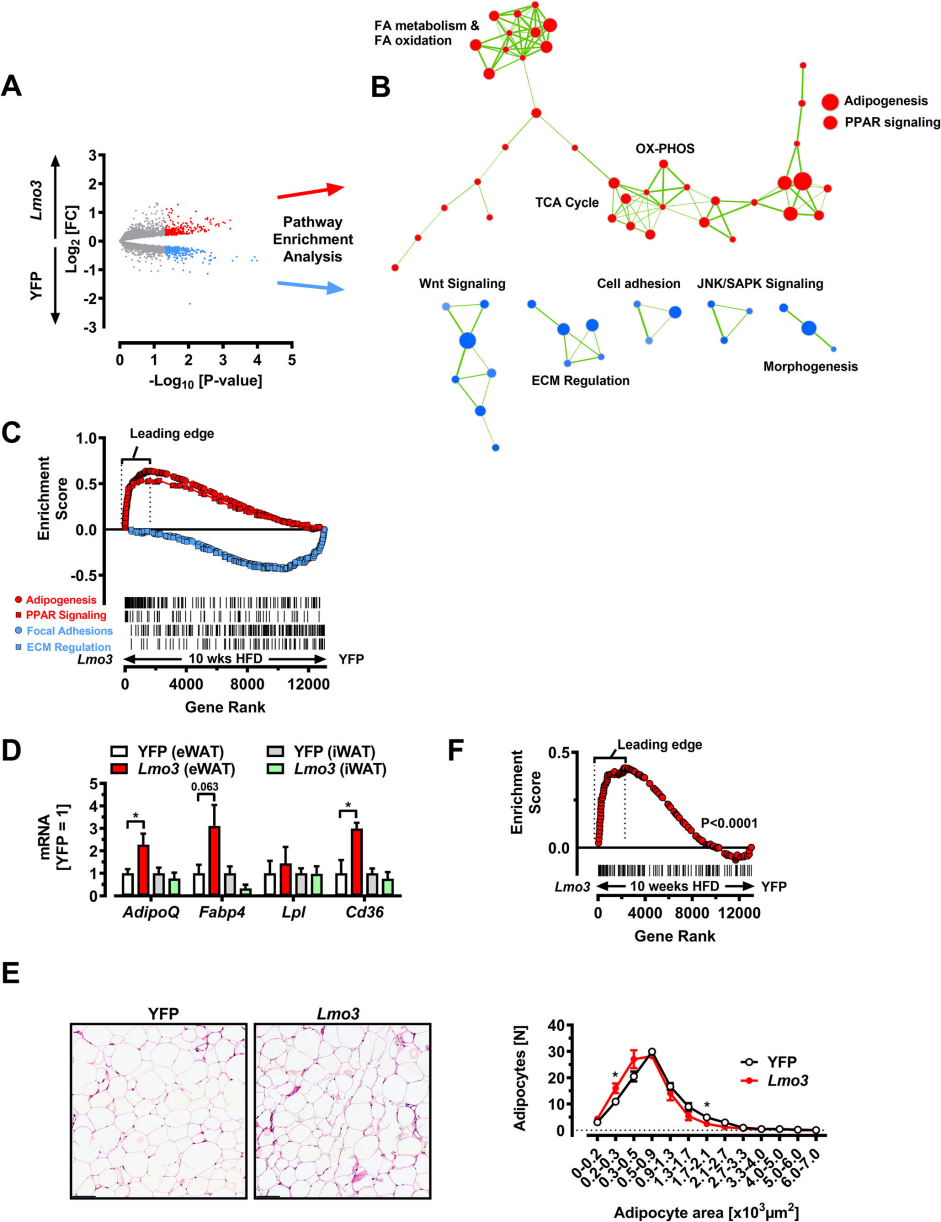
LMO3 targets PPARγ activity in eWAT during obesity. All data are derived from mice on HFD for 10 weeks following transduction of eWAT with either rAAV-YFP or rAAV-*Lmo3*. **A** Log2 fold changes in gene expression data obtained from microarray analysis of eWAT from obese mice (n = 3/group). Red and blue spots correspond to up- and downregulated genes when comparison Lmo3- with YFP gene expression. **B** Cytoscape enrichment map (p-value cutoff: 0.005, FDR Q-value cutoff: 0.1, overlap cutoff: 0.5) of gene set enrichment analysis (GSEA). Gene sets enriched in viWAT from rAAV-*Lmo3* or rAAV-YFP-transduced mice are indicated in red and blue nodes, respectively. Nodes represent gene sets and edges represent mutual overlap. **C** GSEA of eWAT from obese mice. Nominal ES P-value < 0.0001 for “Adipogenesis” & “PPAR signaling” and for “Focal adhesion” and “ECM Regulation,” respectively. Vertical lines for visualization only. **D** Q-PCR analysis in eWAT and iWAT from obese mice of PPARγ target genes (n = 6/group). **E** Left panel: Representative H&E images of eWAT from rAAV-YFP- or rAAV-*Lmo3*-transduced mice. Scale bar 100 μm. Right panel: Quantification of adipocyte size. Total 250–350 cells per group were measured (n = 4/group). **F** GSEA of eWAT from obese mice. Nominal ES P-value < 0.0001 for a custom-gene set derived from human adipose tissue displaying adipose hypertrophy or hyperplasia [31]. The leading edge of this gene set includes genes implicated in lipid droplet growth and regulation of adipocyte morphology including Cidea [58], Pparα [59], and Bnip3 [60]. *p < 0.05, **p < 0.01, ***p < 0.001, ns, not significant

We next sought to clarify the molecular mechanism by which LMO3 promotes the observed metabolic effects. Thus, we analyzed the transcriptome of eWAT from rAAV-YFP and rAAV-*Lmo3* mice using microarrays. Global analysis revealed differential gene expression, with 244 up- and 271 downregulated genes (red representing up- and blue downregulated) in rAAV-*Lmo3* mice, respectively (Fig. 4A and Table S2). GSEA analysis using the MSigDB collection of curated gene pathway annotations yielded enrichment in several gene sets involved in the regulation of adipocyte function (Fig. 4B and Table S3). Gene sets enriched in rAAV-YFP-transduced eWAT of obese mice contained pathways associated with ECM regulation, morphogenesis, and JNK signaling (Fig. 4B and C, and Table S4). *Col6a1*, *Col5a1*, *Col1a1*, *Col4a6*, and *Col2a1* comprised the core enrichment signal of the gene sets “Focal adhesion” and “ECM regulation” among other ECM components (Fig. 4C and Table S4). Among the gene sets enriched in rAAV*-Lmo*3-transduced eWAT, “Adipogenesis” and “PPAR signaling” gene sets, both of which have important roles in metabolic homeostasis, were significantly enriched in rAAV-*Lmo3*-transduced eWAT, as depicted in Fig. 4C and Table S3. This finding is in good agreement with our previous report demonstrating that LMO3 elevates PPARγ signaling during adipogenesis, via blocking (ERK-induced) phosphorylation at serine 112 [3]. Of note, the mRNA levels of *bona fide* PPARγ target genes including *AdipoQ*, *LPL*, and *CD36* were elevated in rAAV-*Lmo3*-transduced eWAT as compared with rAAV-YFP (control) eWAT but remained unaffected in subcutaneous fat, which was not targeted by our AAV (Fig. 4F). Histological examination of eWAT revealed a significant higher number of small adipocytes in eWAT from rAAV-*Lmo3*-transduced mice, as compared with rAAV-*Yfp* controls (Fig. 4G). Furthermore, we found a significant enrichment of genes previously linked to a hyperplastic adipose tissue phenotype [31] in eWAT from rAAV-*Lmo3*- vs. rAAV-*Yfp*-transduced mice (Fig. 4H). Taken together, Lmo3 targets PPARγ in eWAT following HFD.

### Lmo3 increases mitochondrial oxidative capacity

**Fig. 5:**
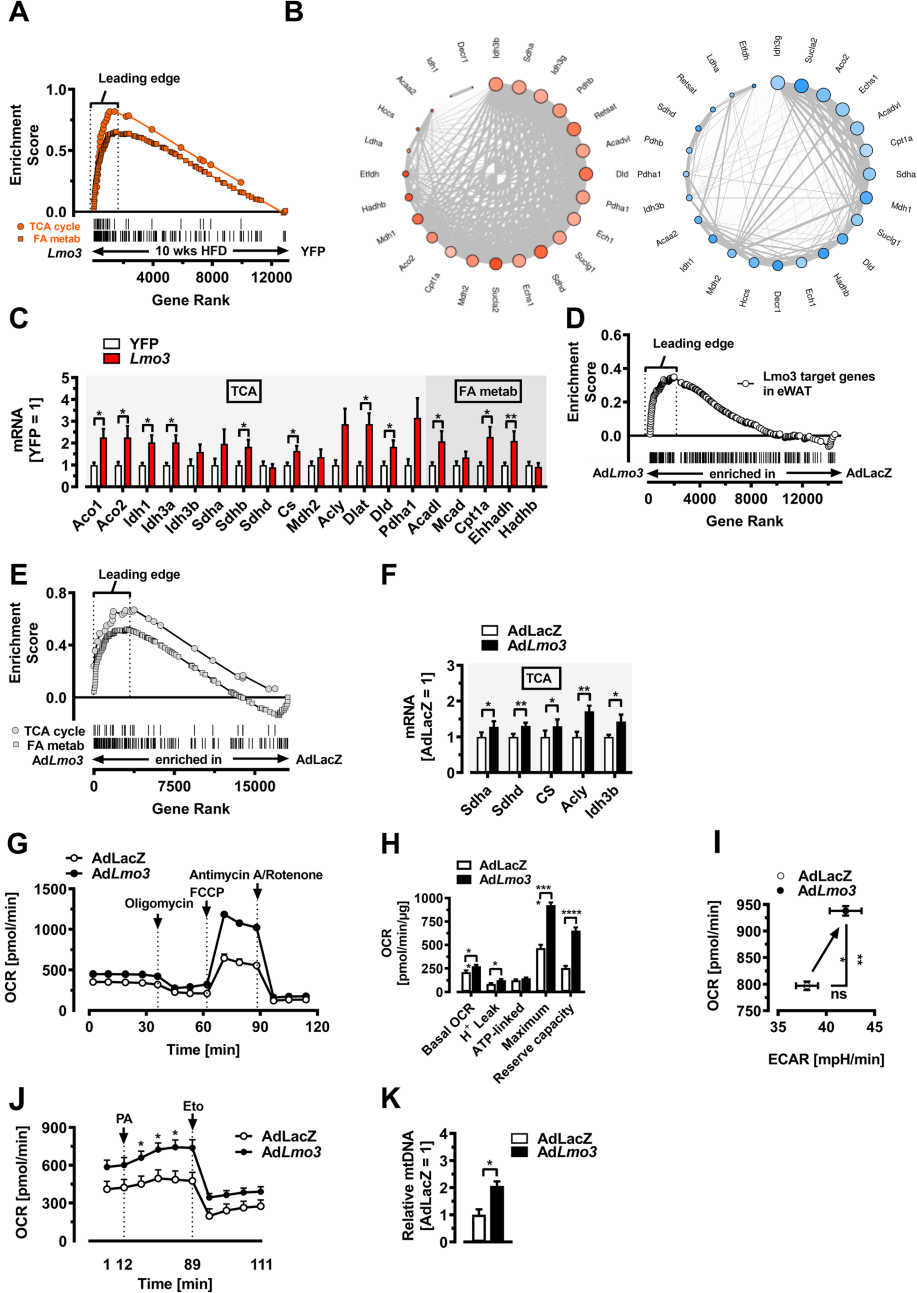
LMO3 increases adipocyte mitochondrial oxidative capacity. eWAT data displayed in (**A**) to (**C**) were from mice on HFD for 10 weeks following transduction of eWAT with either rAAV-*YFP* or rAAV-*Lmo3*. **A** GSEA of eWAT from obese mice. Nominal ES P-value < 0.0001 for “TCA cycle” and “FA metabolism.” Vertical lines for visualization only. **B** Network plots for 24 genes common to the “Leading edge” of OX-PHOS, TCA-cycle, and FA metabolism from the BioCarta, KEGG, and GO compendium. Blue node color represents downregulation and red node color represents upregulation of gene expression in eWAT. The node size is associated with the gene’s co-expression in the entire dataset. The edge (line) thickness is linked to the gene’s connectivity (co-expression within the module). **C** Q-PCR analysis in eWAT from obese mice of selected genes from “TCA cycle” & “FA metabolism” gene sets analyzed by GSEA as shown in (**A**) (n = 8/group). **D** GSEA of Ad*LacZ*- or Ad*Lmo3*-transduced mature 3T3-L1 adipocytes for a custom-gene set featuring 212 rAAV-*Lmo3*-induced genes (Fig. 4A and Supplemental Table; nominal ES P-value < 0.0001). Vertical lines for visualization only. **E** GSEA of AdLacZ- or Ad*Lmo3*-transduced 3T3-L1 adipocytes. Nominal ES P-value < 0.0001. **F** Q-PCR analysis in AdLacZ- or Ad*Lmo3*-transduced 3T3-L1 adipocytes of selected genes from the “TCA cycle” gene set analyzed by GSEA shown in (**E**) (n = 5/group). **G** Oxygen consumption rates (OCRs) of AdLacZ- and Ad*Lmo3*-transduced 3T3-L1 adipocytes (n = 11). **H** OCR and mitochondrial function parameters of Ad*LacZ* and Ad*Lmo3* 3T3-L1 adipocytes (n = 11). **I** OCR and extracellular acidification rates (ECAR) of Ad*LacZ*- and Ad*Lmo3*-transduced 3T3-L1 adipocytes (n = 11). **J** OCR kinetics in AdLacZ and Ad*Lmo3* 3T3-L1 adipocytes after sequential injection of palmitate (PA; 10 μM) and etomoxir (Eto, 100 mM) (n = 5). **K** Q-PCR analysis of mitochondrial DNA content in AdLacZ- or Ad*Lmo3*-transduced 3T3-L1 adipocytes (n = 3). *p < 0.05, **p < 0.01, ***p < 0.001, ns, not significant

To further link the improved glucose tolerance and insulin sensitivity seen in obese rAAV-*Lmo3*-transduced mice with specific molecular alterations, we searched specifically for enriched gene sets implicated in (adipocyte) metabolism. Intriguingly and unexpectedly, we found three top-scoring MgSigdb gene sets “TCA cycle,” “Fatty acid metabolism,” and “OX-PHOS” (mitochondrial oxidative phosphorylation), to be enriched specifically in rAAV-*Lmo3*-transduced eWAT (Fig. 5A and Tables S5), which raises the possibility that LMO3 in eWAT protects against obesity-induced metabolic derangements by increasing mitochondrial functionality in adipocytes. Further, the common genes from the “Leading Edge” of TCA cycle, FA-metabolism, and OX-PHOS gene sets are enriched in a gene co-expression network specifically in *Lmo3*- but not YFP-transduced eWAT (Fig. 5B), further suggesting increased mitochondrial activity in this adipose tissue. In rAAV-*Lmo3*-transduced eWAT of obese HFD-fed mice, the core enrichment signals of the “TCA cycle” and “FA metabolism” gene sets were driven by upregulation of fatty acid mobilization genes including TCA members *Aco1*, *Aco2*, *Idh1*, *Idh3a*, *Sdhd*, *Dlat*, and *Cs*, as well as fatty acid oxidation genes *Acadl*, *Ehhadh* and the mitochondrial free fatty acid transporter *Cpt1a*, which encodes for a member of the carnitine/choline acetyltransferase family, the rate-controlling enzyme of the long chain fatty acid β-oxidation [32]. This was further verified by Q-PCR (Fig. 5C). Notably, an increased activation of these enzymes has been shown both in vitro and in vivo in response to PPARγ agonist treatment, under certain conditions [33, 34]. To determine if adipocyte cell-autonomous effects are responsible for the observed metabolic improvements seen in mice with rAAV-*Lmo3*-transduced eWAT, we turned to mature 3T3-L1 adipocytes engineered to overexpress LMO3. We next asked if the genes regulated in eWAT (Fig. 4A) are controlled by LMO3 in a cell-autonomous fashion. To do so, we built a custom gene set composed of 224 genes that were found to be induced by LMO3 rAAV-Lmo3-transduced eWAT. Next, we performed GSEA on the RNA-Seq dataset from *AdLacZ*- and Ad*Lmo3*-infected 3T3-l1 mature adipocytes using this custom Lmo3 target gene set. Strikingly, we found the rAAV-*Lmo3* gene signature strongly enriched in LMO3-overexpressing 3T3-L1 adipocytes (Fig. 5D), suggesting that the gene regulation following rAAV*-Lmo3*-transduction of eWAT is largely adipocyte cell-autonomous. Of note, GSEA analysis yielded enrichment in “TCA cycle” and “Fatty acid metabolism” gene sets (Fig. 5E), as seen in rAAV-*Lmo3*-transduced mice, further confirming cell-autonomous effects of LMO3. Several mRNA members from the leading edge of the “TCA cycle” and “Fatty acid metabolism” gene sets, including *Sdha*, *Sdhd*, *CS*, *Acly*, *and Idh3b*, were found elevated as a consequence of LMO3 expression in 3T3-L1 adipocytes (Fig. 5F), suggesting that the observed eWAT transcriptome changes are indeed adipocyte-autonomous. Although white adipocytes have considerably less oxidative capacity than brown adipocytes, several reports have demonstrated the importance of white adipocyte mitochondria for glucose homeostasis in mature adipocytes [34–37]. Because several gene sets implicated in mitochondrial functionality were highly enriched in rAAV-*Lmo3*-transduced eWAT (Fig. 2G–I), we further investigated whether *Lmo3* overexpression affects adipocyte mitochondrial oxidative capacity in vitro. Assessment of oxygen consumption rates (OCR) revealed distinct and exclusive increases in mitochondrial respiration in LMO3-expressing 3T3-L1 adipocytes (Ad*Lmo3*) (Fig. 5G, H, and I). Moreover, the administration of exogenous free fatty acids (FFAs) in the form of palmitate resulted in an increase in the OCR in LMO3-expressing 3T3-L1 adipocytes (Fig. 5J). Notably, treatment with etomoxir (a specific inhibitor of Cpt1) was able to restore the OCR to basal levels in control cells, indicating that the increase in the OCR of LMO3-expressing adipocytes after palmitate injection is linked to fatty acid oxidation (Fig. 5J). Quantification of mitochondrial DNA indicated elevated mitochondrial content in LMO3-expressing 3T3-L1 adipocytes (Ad*Lmo3*) as compared with control (AdLacZ) adipocytes (Fig. 5K). These findings implicate LMO3 in control of metabolic state, and in particular driving fatty acid oxidation in mature adipocytes during obesity. Together, our data show that LMO3 expression in eWAT significantly improves glucose clearance in diet-induced obesity and that these effects may be due to increased glucose uptake and mitochondrial functionality by mature adipocytes.

### LMO3 requires NCoA1 to promote mitochondrial oxidative gene expression and glucose uptake

**Fig. 6:**
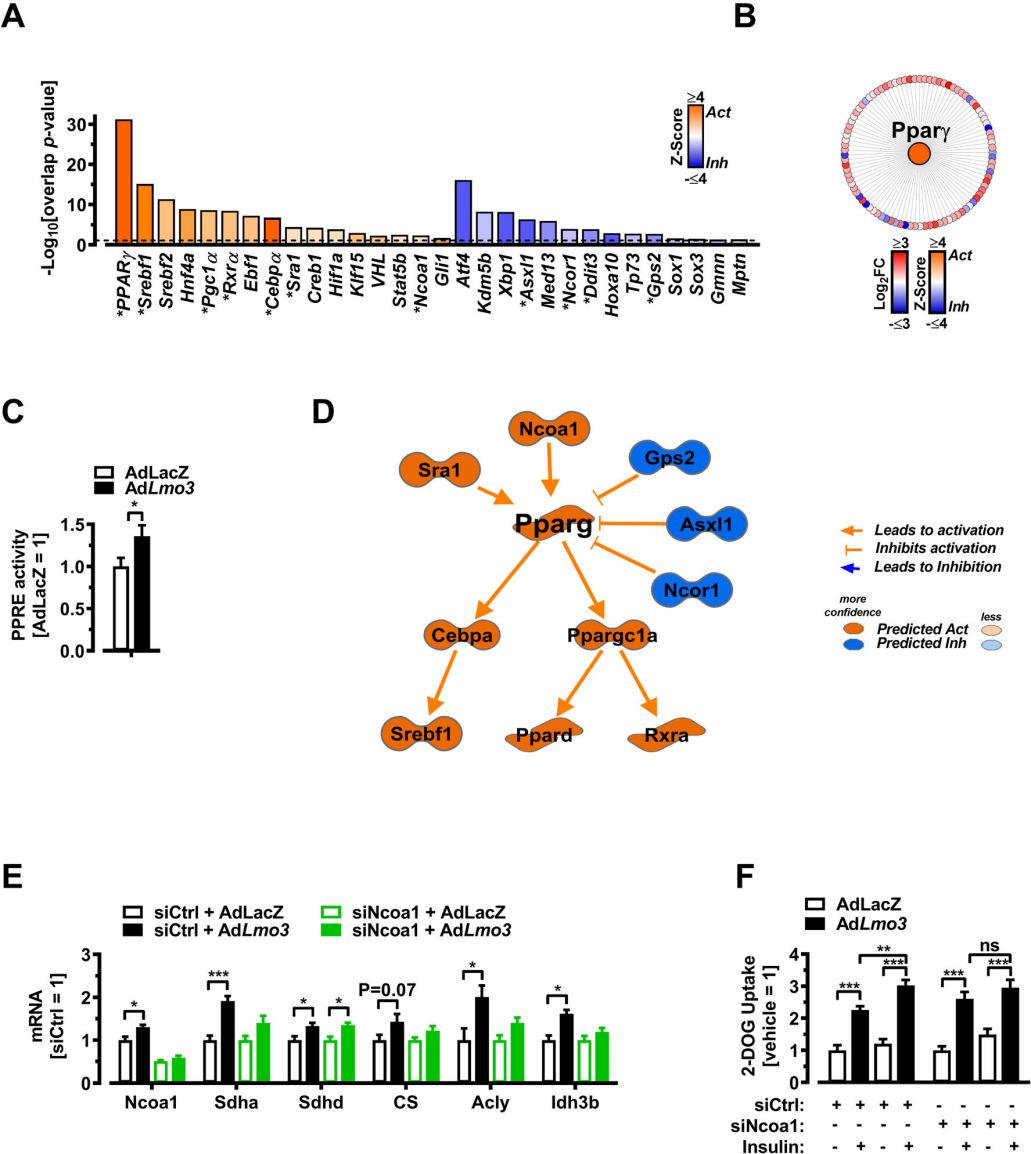
LMO3 increases mitochondrial oxidative gene expression and glucose uptake via NCoA1/SRC-1. **A** IPA-predicted upstream regulators from Ad*Lmo3*- versus AdLacZ-transduced 3T3-L1 adipocytes showing activation Z-score (bars). Top-15 transcriptional regulators with an overlap p < 0.05 by IPA (see “Methods” for description) were predicted to be upstream regulators. *indicates genes comprising the IPA-predicted mechanistic network displayed in (**D**). **B** IPA-predicted upstream regulators (center, colored by Z-score) and target genes (outer circle, colored by Fold Change) in Ad*Lmo3*- versus AdLacZ-transduced 3T3-L1 adipocytes. **C** ELISA-based PPARγ transcription reporter assay demonstrating that LMO3 activated PPARγ transcriptional activity in mature adipocytes compared with LacZ controls (n = 6). **D** IPA-predicted mechanistic network based on upstream regulators as shown in (**F**). See supplemental information for gene symbol description. See Figure S1B for explanation of molecule shapes. **E** Q-PCR analysis of selected “TCA cycle” genes in siCtr- or siNcoa1-transfected control (AdLacZ-) or LMO3-overexpressing (Ad*Lmo3*) 3T3-L1 adipocytes (n = 5). **F** Glucose uptake in in siCtr- or siNcoa1-transfected control (AdLacZ-) or LMO3-overexpressing 3T3-L1 adipocytes (n = 5). After 4-h serum starvation, the cells received mock or insulin treatment for 20 min for measurement of 2-DG uptake. *p < 0.05, **p < 0.01, ***p < 0.001, ns, not significant

To understand which transcriptional/molecular mechanism(s) might account for the effects of Lmo3 on mitochondrial oxidative activity (Fig. 5) and glucose metabolism (Fig. 2), we re-analyzed our RNA-Seq data obtained from LMO3-overexpressing adipocytes in vitro (Fig. 1) and performed “upstream analysis” on the 1093 genes regulated by LMO3 using IPA (Fig. 6A and Table S6). *Pparγ* scored the most significant “upstream regulator,” predicted to be activated by LMO3 overexpression, regulating 93 genes with most of them being induced by LMO3 (Fig. 6A and B), further corroborated by higher levels of nuclear PPARγ isolations binding to a synthetic PPAR response element (Fig. 6C), in line with our previous data demonstrating that LMO3 augments PPARγ activity [3]. In support of LMO3 acting as a glucocorticoid amplifier and PPARγ fine-tuner in adipocytes, the LMO3-induced gene signature was enriched in glucocorticoid and PPARγ activators including dexamethasone (a synthetic glucocorticoid) as well as the PPARγ ligands Troglitazone, Pioglitazone, and Rosiglitazone (as the strongest known agonist of PPARγ enhancing its transcriptional activity on target genes) [38] (Supplemental Figure S3). At the mechanistic level, IPA predicts a molecular network orchestrated by LMO3 and centering around PPARy (Fig. 6D), acting upstream of *Pgc1α* and *Cebpα*. Importantly, upstream of PPARγ, this network features several known positive and negative regulators of PPARγ activity including *Ncoa1*, *Sra1* and *Ncor1*, *Asxl1*, and Gps2, respectively (Fig. 6D). A potential mechanism linking mitochondrial oxidative activity and glucose metabolism may involve recruiting NCOA1, predicted to be more active upon in LMO3-overexpressing cells (Fig. 6D). Recruitment of NCOA1 (also known as steroid receptor coactivator 1; SRC-1) to PPARγ has been shown to increase glucose uptake into 3T3-L1 adipocytes [10] and promote mitochondrial oxidative gene expression and fatty acid oxidation in brown fat [12]. To investigate if NCOA1 is required for the increased mitochondrial oxidative gene expression and glucose uptake observed in *Lmo3*-overexpressing cells, we silenced NCOA1 expression in mature adipocytes by RNAi (Supplemental Figure S3C and D). *Lmo3* expression resulted in increased *Ncoa1* mRNA expression, not seen when adipocytes were treated with a siRNA targeting *Ncoa1* (Fig. 6E). In addition, LMO3 was unable to increase the expression of the mitochondrial genes *Sdha*, *CS*, *Acly*, and *Idh3b* when *Ncoa1* was silenced (Fig. 6E), without affecting differentiation levels between these groups (Supplemental Figure S3E). Furthermore, LMO3 was unable to increase insulin-stimulated glucose uptake in the presence of a siRNA targeting *Ncoa1* (Fig. 6F). Thus, LMO3 orchestrates a transcriptional program potentially rewiring mature adipocyte glucose and mitochondrial metabolism via fine-tuning PPARγ activity.

### LMO3 promotes mitochondrial oxidative capacity in human mature adipocytes

**Fig. 7:**
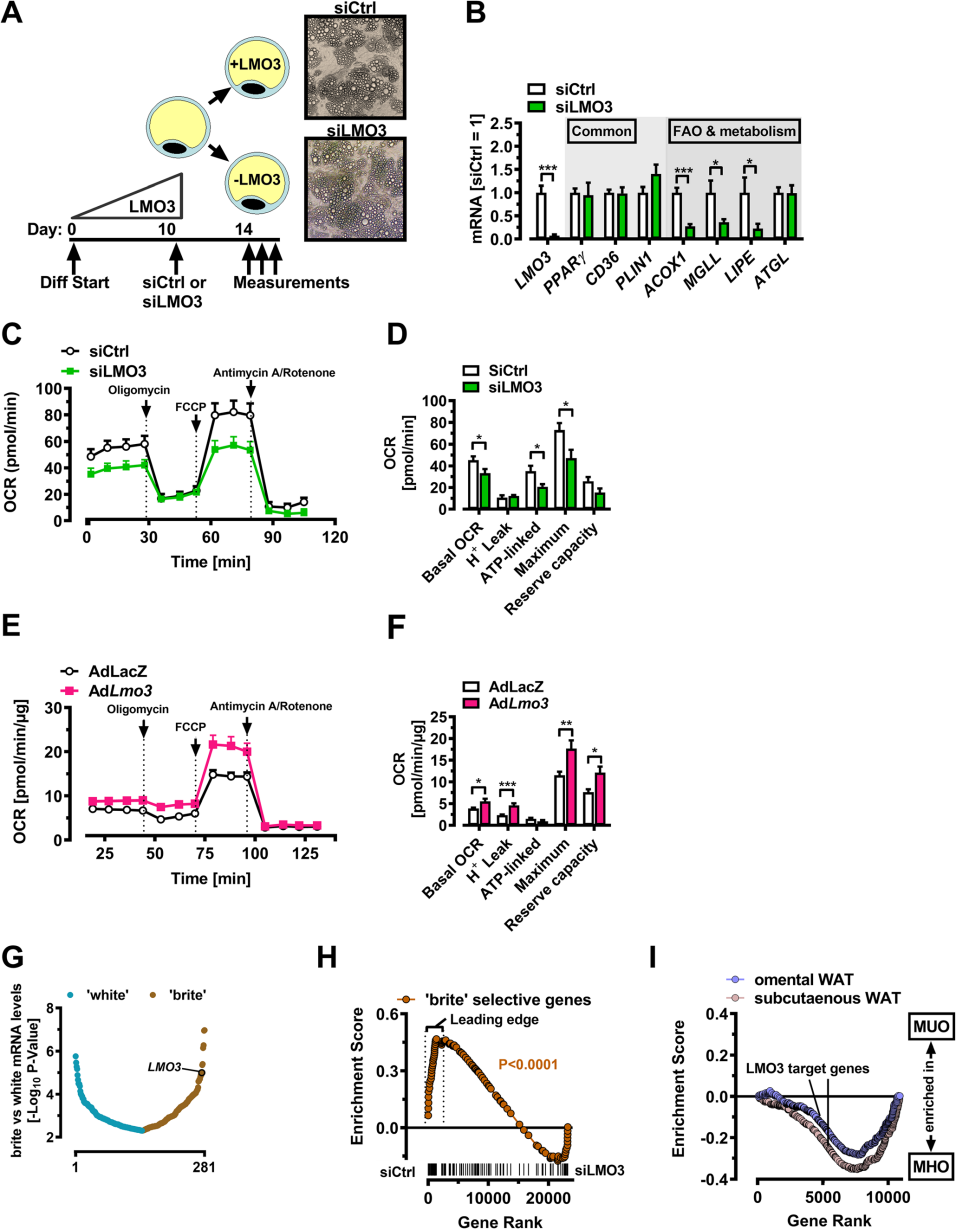
LMO3 promotes mitochondrial oxidative capacity in human mature adipocytes. **A** Experimental scheme for silencing LMO3 in human mature adipocytes. Photomicrographs show mature adipocytes 4 days after siRNA transfection. **B** Q-PCR analysis of selected genes from siCtrl or siLMO3-transfected human mature adipocytes (n = 5–7). **C** OCR of siCtrl- or siLMO3-transfected human mature adipocytes (n = 8–11). **D** OCR and mitochondrial function parameters of siCtrl- or siLMO3-transfected human mature adipocytes (n = 8–11). **E** OCR of AdLacZ- or Ad*Lmo3*-transduced human mature adipocytes (n = 11). **F** OCR and mitochondrial function of AdLacZ- or Ad*Lmo3*-transduced human mature adipocytes (n = 11). **G** Rank order of 281 human white adipocyte genes correlating with forskolin treatment (forskolin vs. vehicle, P-value < 0.005) obtained from public data [41]. **H** GSEA of genes induced in “brite” adipocytes [41] in siCtrl- versus siLMO3-transfected mature human adipocytes. P-value of the Nominal Enrichment Score for the indicated gene set is indicated. **I** GSEA of adipocyte LMO3 target genes in WAT derived from metabolically “healthy” (MHO) or “unhealthy” (MUO) morbidly obese patients based on HOMA-IR [42] using a custom gene set composed of LMO3-regulated genes in human mature adipocytes. Note that LMO3-induced genes are enriched in MHO patients. NES P-value < 0.0001 for both, omental and subcutaneous WAT. *p < 0.05, **p < 0.01, ***p < 0.001, ns, not significant

Next, we asked if LMO3 exerts similar function also in human adipocytes, which strongly express LMO3 protein [3]. Silencing of LMO3 at the onset of differentiation results in diminished adipogenesis [3]. To avoid indirect effects arising from different levels of differentiation, we chose to *first* de-novo differentiate hASC isolated from subcutaneous lipoaspirates into mature adipocytes, followed by transfection with our established siRNA molecules (Fig. 7A). We observed no evident changes in cell morphology 3–4 days post-transfection (see photomicrographs in Fig. 7A) but found a significant reduction in LMO3 mRNA expression, along with several fatty acid mobilization genes including *ACOX1*, *MGLL*, and *LIPE*, while common adipocyte genes (*CD36*, *PLIN1*, and *PPARγ*) were unaffected (Fig. 6B). Silencing of LMO3 expression in human mature adipocytes reduced mitochondrial oxidative capacity including basal and maximal OCR (Fig. 7C and D), while LMO3 overexpression by adenoviral transduction strongly promoted mitochondrial oxidative capacity (Fig. 7E and F), which is in good agreement with our data in murine 3T3-L1 cells. Since the gene programs selective for “brite” adipocytes are associated with increased mitochondrial function and energy consumption [39], it is plausible that LMO3 might be part of a “molecular switch” for brite adipocytes. Chronic treatment of human and rodent white adipocytes in vitro with a PPARγ ligand such as rosiglitazone or a cAMP inducer such as forskolin induces a phenotypic switch which results in browning of white adipocytes (the so-called brite adipocyte) [39–41]. Interestingly, upon re-examination of a recently published dataset [41], we identified *LMO3* as a top-scoring gene in human adipocytes upon acquisition of a “brite” phenotype (Fig. 7G). To investigate if LMO3 might contribute *functionally* to a brite adipocyte molecular network, we investigate if loss of *LMO3* gene expression in human adipocytes affects expression of a brite adipocyte gene signature [41]. To do so, we first profiled genome-wide expression changes that occur in response to silencing LMO3 expression, comparing patterns between siCtrl- and siLMO3-treated human mature adipocytes, yielding 947 genes differentially regulated more than ± 1.5-fold following LMO3 knockdown (Table S6). Using this LMO3 gene signature, GSEA analysis showed that a brite-selective gene program was enriched in LMO3-expressing (siCtrl) human mature adipocytes, absent in LMO3 knockdown (siLmo3) adipocytes (Fig. 6H). To begin to understand if the altered adipocyte metabolism observed in LMO3-expressing adipocytes in vitro may also be associated with an “healthier” AT in obesity, we re-analyzed a previously published microarray dataset of WAT derived from morbidly obese patients grouped into “metabolically healthy” or “metabolically unhealthy” obesity (MHO vs. MUO) based on the degrees of insulin sensitivity [42]. Specifically, we asked if the *LMO3*-dependent genes are preferentially expressed in metabolically healthy WAT or distributed equally among these 2 states (i.e., HOMA-IR < 2.4 or ≥ 2.4, respectively; [42]). We thus performed GSEA using a custom gene set composed of the genes differentially expressed between siCtrl- and siLmo3-transfected human mature adipocytes. Surprisingly, genes expressed at higher levels with endogenous LMO3 expression (siCtrl) were highly significantly enriched in metabolically healthy omental as well as subcutaneous WAT in obese patients (Fig. 7I). Taken together, these results demonstrate that LMO3 drives a gene program in human and murine adipocytes promoting mitochondrial function.

## Discussion

In the present study, we have shown that LMO3 increases insulin-induced glucose uptake and GLUT4 translocation, along with enhanced mitochondrial oxidative capacity in adipocytes. In vivo, we demonstrate that LMO3 preserves a metabolically beneficial visceral adipose tissue expansion and insulin sensitivity along with enhanced adiponectin secretion during obesity. At the molecular level, LMO3 increased PPARγ activity as well as NCoA1 expression, required for glucose uptake and mitochondrial oxidative gene expression. Our data expand greatly upon our previous studies of LMO3 function during adipogenesis to illuminate the role of this protein in mature fat during obesity.

In our study, overexpression of LMO3 in 3T3-L1 adipocytes has no influence on proximal insulin signaling, as insulin-stimulated Akt activation remains intact. Since insulin-activated PI3K-Akt induces GLUT4 translocation to the plasma membrane, which we show is elevated in LMO3-expressing adipocytes, how does LMO3 enhance insulin-induced GLUT4 translocation? PI3K-Akt activation is required but not sufficient for insulin-induced GLUT4 translocation [43]. GLUT4 translocation requires the intersection of insulin signaling (PI3K-Akt) and vesicle trafficking pathways [44, 45]. Thus, the increased GLUT4 translocation seen in LMO3-expressing adipocytes may be explained by regulation of the cytoarchitecture involved in GLUT4 translocation and targeted by LIM domains [25] rather than PI3K-Akt-insulin signaling. A similar scenario exists for other proteins regulating insulin-induced GLUT4 translocation including Rab10 [46], Tmod3 [26], Usp25m [47], the atypical PKC ζ/λ isoform [48], and a signaling pathway involving CAP/Cbl/TC10 [43].

We previously determined that LMO3 boosts PPARγ activity by attenuating its phosphorylation at Serine 112 [3], which increases its transcriptional activity [49, 50]. In line with these data, using transcriptional profiling as well as biochemical assays in eWAT complemented by LMO3 gain-of-function 3T3-L1 adipocytes, we here show that LMO3 affects mature adipocyte function via PPARγ during obesity based on the following observations: (i) selective enrichment of gene sets implicated with PPARγ activity, (ii) IPA revealed that many of the > 600 LMO3-increased genes in 3T3-L1 adipocytes were predicted to be PPARγ targets, (iii) increased serum levels of adiponectin, and (iv) cell-autonomous increase in adipogenic and PPAR-signaling genes in mature adipocytes. Another possible mechanism affecting PPARγ tone may involve the PPARγ coactivator NCOA1, which is increased by LMO3. NCOA1 associates with PPARγ leading to increased transactivation of those PPARγ target genes that are involved in glucose homeostasis [9]. Our observations that glucose uptake as well as expression of a subset of PPARγ dependent genes require (LMO3-induced) *Ncoa*1 expression, at least in part, favor such a mechanism. Future experiments using PPARγ null cells shall clarify the role of the LMO3/NCOA1 axis on regulation of PPARγ dependent phenotypes.

PPARγ is also a major driver of the genomic reprogramming during “browning” of white adipose tissue and the majority of PPARγ functions are common between white and “beige/brite” adipocytes [39, 40]. Further studies will shed light on the possibility that LMO3 reprograms PPARγ towards brite-selective PPARγ-binding sites within white adipocyte chromatin. MHO has recently been associated with increased expression of TCA cycle and OXPHOS genes in adipose tissue indicating improved mitochondrial function [51]. Although it is still controversial if adipose mitochondrial dysfunction is a cause or consequence of insulin resistance [52], mitochondrial remodeling in white fat following rosiglitazone treatment suggested that enhanced lipid utilization in this tissue may affect whole-body energy homeostasis and insulin sensitivity [34]. Likewise, increasing mitochondrial respiration through the fat tissue-specific deletion of the mitochondrial transcription factor TFAM increases systemic glucose tolerance [53]. Since adipose tissue from MHO subjects is enriched in LMO3-dependent genes (Fig. 6I), and LMO3 overexpression in vivo and in vitro augmented TCA cycle and OXPHOS gene expression (as seen in MHO adipose tissue), LMO3 may thus contribute to the preserved insulin sensitivity characterizing MHO. Functionally, these molecular changes were apparent in the enhanced capacity of LMO3-expressing 3T3-L1 cells to consume oxygen and oxidize palmitate (Fig. 5). Thermogenesis from activated brown adipocytes and/or “beige/brite” adipocytes residing within subcutaneous white adipose tissue can act as a “metabolic sink” for glucose, free fatty acids, and other metabolite and contribute to the observed improvement in insulin sensitivity [54]. Elevated serum adiponectin in LMO3-overexpressing mice might contribute to the differences in basal glucose levels since adiponectin strongly suppresses hepatic gluconeogenesis by inhibiting genes involved in glucose production [55, 56]. The mRNA levels of key gluconeogenic genes phosphoenolpyruvate carboxykinase (PEPCK) and G6Pase in the rAAV-Lmo3 injected mouse livers were indeed lower than corresponding levels in livers from control (rAAV-YFP injected) HFD-fed animals (data not shown), suggesting, albeit indirectly, that hepatic glucose output via gluconeogenesis might be reduced in rAAV-Lmo3 targeted mice; however, this speculation awaits experimental proof with future experiments. A similar mechanism was reported for the ARF family of small GTPases ARFRP1 genetically expressed in vivo specifically in mature adipocytes [57]. But are visceral adipocytes also capable of thermogenesis and if so, what is their contribution to nutrient homeostasis during obesity? A recent report demonstrated the existence of “classical” UCP1^+^ as well as UCP1^−^ thermogenic adipocytes even in the visceral (murine) depot [58]. Functionally, thermogenic (UCP1^+^) visceral adipocytes, e.g., generated through deletion of the transcriptional regulator Zfp423 specifically in *visceral* adipose tissue, were capable of improving insulin sensitivity and glucose tolerance in obese mice, without impacting body weight [59]. Thus, an LMO3-induced shift of visceral adipocytes towards a thermogenic phenotype over time might represent a thermogenic effect capable of modulating nutrient homeostasis. Whether LMO3 is also a suitable therapeutic target remains to be demonstrated.

## Methods

### Mice

C57BL/6J male mice were purchased from The Jackson Laboratory. Animal experimentation conformed to protocols approved by the Institutional Animal Ethics Committees of the Medical University Vienna and studies using cells isolated from human donors have been approved by the ethics committee of the Medical University of Vienna. Mice were given free access to food and water. High-fat diet was from Research Diets (D12492). Standard diet was from Harlan Laboratories (2018S). Animals were exposed to HFD or standard diet at an age of 6 weeks.

### rAAV vector construction and packaging

The rAAV plasmid contains a vector expression cassette consisting of the CMV enhancer and chicken β-actin (CBA) promoter, woodchuck post-transcriptional regulatory element (WPRE), and bovine growth hormone (bGH) poly-A flanked by AAV inverted terminal repeats. Transgenes encoding YFP or LMO3 were inserted into the multiple cloning sites between the CBA promoter and WPRE sequence. The engineered hybrid serotype Rec2 vector were packaged and purified as previously described [22].

### rAAV injection to epididymal WAT

Animals aged 8 weeks were anesthetized using isoflurane (1–3% in oxygen, O_2_ flow rate: 2 L/min) in combination with buprenorphine (0.06 mg/BW Temgesic subcutaneously, RB Pharmaceutical Limited, Berkshire, UK) and animals’ corneas were protected with an eye ointment to prevent corneal drying and damage. Once anesthesia was fully induced, the animal was shaved in the epididymal abdominal area and the skin was sterilized with betaisodona followed by a 1.5 cm midline incision (linea alba). Thereafter, the epididymal fat pad was grabbed and pulled out gently with forceps and the rAAV was injected at multiple sites (1.0 × 10^10^ vg per 20 μl in phosphate-buffered saline) with a 0.3 cc, 31 G insulin syringe. After ensuring no virus leaked during the injection, the fat pad was gently forced back into the abdominal cavity and the procedure was repeated on the opposite eWAT to complete the bilateral injection. The peritoneal layer was sutured with a continuous suture pattern (absorbable suture, Vicryl 5.0, Johnson & Johnson), the skin was closed with stainless steel wound clips (Leica Biosystems), and the wound was coated with mercuchrome-iodine solution or blue spray. For postoperative rehydration, mice were injected with 4 μl/g BW 0.6% NaCl subcutaneously and mice were allowed to recover under a warming lamp before they were transferred back to their cages.

### Adenovirus experiments

A codon-optimized murine Lmo3 gene sequence (Gene ID: 196843) was cloned from pJ201 plasmid (DNA2.0) into an adenoviral expression vector (pAdlox) using restriction enzymes BamHI and EcoRI (NEB). 3T3-L1 adipocytes were infected with 100 pfu/cell on day 7 of differentiation. Experiments were performed 3 days after adenoviral transduction. Lmo3 (AdLmo3) and LacZ (AdLacZ) expressing viruses were produced according to published protocols (Hardy et al., 1997). In brief, Sfi I-digested Adlox plasmid DNA was cotransfected with psi5 DNA into Cre8 cells using FuGENE 6 Transfection Reagent (Roche). Three days after transfection, cells were collected by centrifugation and recombined viruses extracted from cell pellets by four freeze and thaw cycles. Cell debris was removed by centrifugation. HEK293 cells (ATCC) were used to amplify adenoviral particles. Amplified AdLmo3 and AdLacZ were purified by cesium chloride density-gradient ultracentrifugation, collected from the gradient, diluted 1:1 in 2× storage buffer (10 mM Tris pH 8.0, 100 mM NaCl, 0.1% BSA, 50% glycerol), and stored in small aliquots at −20 °C.

### Knock-down experiments

For a transient transfection approach with the aim to silence Ncoa1 expression, de-novo differentiated mature 3T3-L1 adipocytes were transfected using the RNAiMax Lipofectamine Transfection reagent (Invitrogen) according to the manufacturer’s instructions. A specific “*Silencer*” Ncoa1 siRNA and together with a negative control siRNA (cat. No.: AM4611) were commercially purchased (Invitrogen, see Table S8 for the oligonucleotide sequence).

### Glucose and insulin tolerance tests

Following an overnight fast, mice were administered glucose (1 g/kg) by oral gavage, and blood samples for glucose and insulin determination were collected from the tail vein at the indicated times. Insulin tolerance was assessed after a 2 h fast by intraperitoneal administration of human regular insulin (0.75 U/kg) and blood glucose monitoring. Glycemia was assessed using an Accu-Chek (Roche) glucometer. Plasma insulin levels were determined using the Ultrasensitive Mouse Insulin ELISA kit (Mercodia).

### Glucose uptake

Glucose uptake was measured as the incorporation of radiolabeled 2-deoxyglucosein day 7 3T3-L1 differentiated adipocytes. Briefly, 2 × 10^5^ cells per well were grown in 12-well plates and serum-starved for 16 h. The cells were then washed twice with Krebs-Ringers Henseleit (KRH) buffer (20 mM HEPES pH 7.4, 136 mM NaCl, 4.7 mM KCl, 1.25 mM MgSO_4_, and 1.25 mM CaCl_2_) containing 0.1% bovine serum albumin (BSA) and incubated for 15 min in 0.45 ml KRH/BSA, followed by addition of 0.1 μM cold 2-deoxy-D-glucose and 0.5 μCi 2-deoxy-D-[3H]-glucose (Perkin Elmer) for 4 min. Following the incubation, cells were placed on ice, washed twice with ice-cold PBS, and lysed in 0.5 ml of 0.05% sodium dodecyl sulfate (SDS). The lysate (0.4 ml) was counted in 5 ml of scintillation fluid using a Beckman LS6500 scintillation counter. Rates of non-specific glucose uptake were determined in samples pre-treated for 10 min with cytochalasin B (10 μM, Sigma) and were subtracted from the total uptake. The specific glucose uptake was normalized to protein content**.**

### Clinical chemistry and lipid analysis

At the time of sacrifice, blood was collected via the vena cava into EDTA tubes (MiniCollect® 1 ml K3EDTA Blood Collection Tube, Greiner Bio-One), centrifuged for 10 min at 2000×*g*, and aliquots of plasma were stored at −80 °C for further analyses. Non-esterified fatty acids (NEFAs) in the liver were determined using the NEFA-C kit (ACS-ACOD method; Wako Chemicals, Neuss, Germany). Adiponectin levels were determined by ELISA kit (CrystalChem) according to the manufacturer’s instructions. All measurements were done according to the manufacturer’s protocols using a Benchmark 550 Micro-plate Reader (Bio-Rad, Hercules, CA, USA).

### Gene expression profiling

Total RNA extraction, preparation of terminal-labeled cDNA, hybridization to genome-wide murine or human Gene Level 1.0 ST GeneChips (Affymetrix), and scanning of the arrays was carried out at the Core Facility Genomics of the Medical University of Vienna according to manufacturer’s protocols (https://www.affymetrix.com) as described [3, 60]. Array data were normalized and statistically analyzed as described elsewhere (Klinglmueller et al., 2011). (i) eWAT samples: Each of the six microarray samples represent eWAT isolated from obese rAAV-YFP- or rAAV-*Lmo3*-transduced mice following 12 weeks of HFD (started week 6 of age). (ii) Total RNA isolated from siCtrl- or siLmo3-transfected human mature adipocytes (n = 2 per genotype) was labeled and hybridized to Affymetrix Human Gene Level 1.0 ST Arrays according to manufacturer’s protocol (www.affymetrix.com) and the fluorescence intensities normalized and statistically analyzed as described elsewhere [61]. Statistical significance of the estimated differential effects was assessed using t-tests and corresponding p-values. Multiple testing adjustments were performed using the Benjamini-Hochberg procedure, which provides control of the false discovery rate. (iii) AdLacZ- and AdLmo3-transduced 3T3-l1 adipocytes: Library preparation and RNA-Seq was carried out by the Core facility Genomics, Medical University of Vienna, Vienna, Austria, as described [62]. Briefly, sequencing libraries were prepared using the NEBNext® Ultra™ II RNA Library Prep Kit according to manufacturer’s instructions and sequenced on an Illumina NextSeq500 platform in a 75bp single-read mode. RNA-Seq data were mapped to the Mus musculus/mmc10 assembly of the murine genome using the STAR Aligner [63]. Differential gene expression was analyzed using DESeq2 [64].

### Ingenuity Pathway Analysis

Differentially expressed protein-coding genes (P < 0.05, DESeq2) between Lmo3- or LacZ-expressing 3T3-L1 adipocytes were analyzed for discovery of regulatory networks using Ingenuity Pathway Analysis (IPA, Ingenuity Systems, Inc., Redwood City, CA, USA). Only experimentally observed direct regulatory relationships were considered for network generation. We used the “Upstream Regulator Analysis” tool to identify upstream regulators that may be responsible for the gene expression changes in the dataset. This analysis seeks to identify upstream regulators and predict whether they are activated or inhibited given the observed expression changes of their downstream targets, without taking into account expression of the upstream regulators themselves. We focused our analysis on transcription regulators for which an activation state prediction could be generated, and ranked them based on the p-value generated by IPA for their overlap with the expected causal effects on their targets. We then generated a regulatory network based on the known relationships between the top candidate regulators actually present in the dataset and the known target molecules in the dataset used to identify them.

### Gene set enrichment analysis

Biological insights concerning the differentially expressed genes were explored via gene set enrichment analysis (GSEA). The analysis was performed with the GSEA software [65] (version 2.1.0) using the c2 (version 5) gene set database. In addition, two custom gene sets were used from adipose tissue genes regulated by morphology [31]. P-values and the false discovery rate (FDR) for the enrichment score of each gene set were calculated based on 1000 gene set permutations. Enrichment plots were generated using Cytoscape 3.3.0 and the plugin Enrichment map [66].

### Weighted gene co-expression network analysis

The Pearson correlations were calculated for all pairs of genes in the samples. The resulting correlation matrix was transformed into a matrix of connection strengths by raising the absolute value of the correlation coefficients to the power ß. A ß of 6 was used to satisfy the scale free topology criterion [67]. The top 25% of genes displaying the highest variability over the entire dataset were selected for module detection. From these genes, modules were determined using the “blockwise” module detection function of the weighted gene co-expression network analysis (WGCNA) package with default values except for the minimal module size and the merge cut height parameters (set to 20 and 0.05, respectively).

### Q-PCR

Q-PCR was performed as previously published [3]. All Q-PCR data are normalized to amounts of acidic ribosomal phosphoprotein P0 (RPLP0), unless otherwise stated. Primer sequences are summarized in Supplemental Table S8.

### Western blot analysis

Protein concentration for the resulting lysates was determined using the BCA (bicinchoninic acid) Protein Assay kit from Pierce, and lysates were run on 10% polyacrylamide gels. PVDF membranes (GE healthcare) were incubated with following antibodies: LMO3 (Abnova), GAPDH and PPARγ (Santa Cruz), GFP (Abcam), β-actin (Sigma), p-S476, p-T308, Akt, as well as HRP-conjugated IgG secondary antibodies were used (Cell Signaling) and blots were developed with ECL Plus Western Blotting Detection System (GE Healthcare). ImageLab software (BioRad) was used for densitometric quantification.

### PPARγ transcription factor assay

PPARγ activity was determined with the ELISA-based PPARγ transcription factor assay kit (Abcam, Cambridge, MA, USA) following the manufacturer’s protocol. In brief, 50 μg of nuclear extract from Ad-LacZ- or AdLmo3-transduced 3T3-L1 adipocytes was added to each well of a 96-well plate precoated with a specific double-stranded DNA (dsDNA) sequence containing the peroxisome proliferator response element (PPRE). The wells were incubated overnight at 4 °C without agitation. Specific primary antibody directed against PPARγ was added, followed by HRP-conjugated secondary antibody. PPARγ was detected with sensitive colorimetric readout at 450 nm after the addition of developing and stop solution.

### Confocal immunofluorescence microscopy

All immunofluorescence slides were mounted for imaging with confocal laser scanning microscopy (LSM 700 Carl Zeiss) as follows: Murine eWAT was fixed in 10% formalin (Sigma) then paraffin embedded. Paraffin sections were deparaffinized followed by antigen retrieval by boiling in citrate buffer (Dako). Sections were blocked with 3% bovine serum albumin (BSA) then incubated with: LMO3 (Santa Cruz), Caveolin (Cell Signaling), or respective IgG Control (all Sigma). Additional blocking step with goat serum (Invitrogen) prior to TRITC (Jackson Immunotech) was performed. Thereafter remaining secondary antibodies: Cy5 (Jackson Immunotech) and Alexa 488 (Invitrogen) followed by 4′,6-diamidino-2phenylindole (DAPI, Sigma). All antibodies were diluted in 3% BSA solution.

### Histology and adipocyte size

Hematoxylin and eosin (H&E) staining was performed on 5 μm paraffin sections. Adipocyte size distribution was determined by semi-automated morphometry. In brief, 3 fields of view of 3 different sections per animal were quantified. Visceral fat pads from 4 animals per group were analyzed. Adipocyte size was measured using Adiposoft [68].

### Myc-GLUT4-mCherry translocation

To investigate the consequences of LMO3 on GLUT4 translocation, we used a previous published method [26]. In Brief, 3T3-L1 cells were transduced with lentivirus containing the lentiviral backbone pLenti-myc-GLUT4-mCherry (Addgene, Cat. No. 64049) followed by transduction with AdLacZ or Ad*Lmo3*. In non-permeabilized condition, cells were incubated with primary mouse anti-Myc Ab (Santa Cruz Biotechnology, clone 9E10) followed by Alexa Fluor 488-conjugated anti-mouse secondary Ab. Mounted samples were subjected to confocal imaging. Zeiss software was used for quantitative measurements of GLUT4 translocation. The ratio of surface to total GLUT4 was quantified by detecting surface GLUT4 through anti-Myc fluorescence immunolabeling and total GLUT4 through mCherry fluorescence in non-permeabilized cells. Data in each group were normalized and expressed as a percentage of insulin-treated control cells. Data presented are representative confocal microscopic images and means ± SEM of about 40–100 cells in each group from three independent experiments.

### PPARγ activity assay

Nuclear proteins were extracted from 3T3L1 mature adipocytes with NE-PER Nuclear and Cytoplasmic Protein Extraction Kit (Thermo Scientific); 50 μg of nuclear proteins per sample were used in PPARγ transcription factor assay (ab133101, Abcam, MA, USA). Briefly, specific dsDNA sequence containing the peroxisome proliferator response element (PPRE) was immobilized on bottom of a 96-well plate. Active PPARγ from nuclear extracts binds to PPRE and was detected with primary anti-PPARγ (Phospho-Thr69,71) antibody. A secondary HRP-conjugated antibody was added to provide colorimetric readout at 450 nm. Absorbance was read on Benchmark Plus microplate spectrophotometer (Bio-Rad, PA, USA). The experiment was repeated two times, in triplicate for every sample.

### Isolation of human preadipocytes

Human subcutaneous adipose tissue was obtained from healthy individuals undergoing lipoaspiration as described [3]. A total of 5 female donors were used throughout the study. This study was approved by the Medical University of Vienna’s ethics committee and the General Hospital of Vienna (EK no. 1115/2010). All subjects gave written informed consent prior to taking part in the study.

### Human and murine adipocyte differentiation

hASCs were kept in growth medium until confluent. Two days post-confluent ASC were induced to differentiate by adding a differentiation cocktail for 10 to 13 days as described [3]: DMEM/Ham’s F12, 10% FBS, biotin (33 μM), pantothenic acid (17 μM), triiodothyronine (T3) (1 nM), human insulin (870 nM), troglitazone (TZD) (5 μM), transferrin (1 μg/ml), and for the first 3 days including dexamethasone (1 μM) and isobutyl-methylxanthine (IBMX) (500 μM) (all from Sigma). At 2 days post-confluency, 3T3-L1 cells were treated with a differentiation cocktail containing: DMEM, 10% CS, insulin (870 nM), TZD (5 μM), Dex (1 μM), and IBMX (500 μM). On day 3 of differentiation, cells were switched to differentiation cocktail excluding IBMX and Dex for remaining duration of differentiation.

### Human mature adipocyte transfection

hASCs were induced to differentiate into mature adipocytes for 10 days. Thereafter, siRNA (Invitrogen; 100 nmol/L; [3]) were delivered into mature adipocytes by Amaxa nucleofection (Lonza Bioscience) according to manufacturer’s recommendations. Cells were utilized 48–72 h after transfection.

### Measurement of mitochondrial DNA

Total DNA was extracted from cultured cells using the Qiagen DNA extraction kit. DNA concentrations were determined by NanoDropTM 1000 Spectrophotometer (Thermo Scientific, Wilmington, DE, USA). One ng of total DNA was used to determine the ratio of mitochondrially encoded NADH dehydrogenase 2 ND2 (*mt-Nd2*) to nuclear encoded NADH:Ubiquinone Oxidoreductase Core Subunit V1 (*Ndufv1*) by real-time PCR [69] using the oligos listed under Supplemental Table S8.

### Metabolism assays

ECAR and OCR measurements were made with an XF-24 Extracellular Flux Analyzer (Seahorse Bioscience) as described [62]. In brief, bead-purified visceral APs were seeded into XF 24-well cell culture microplates. After a 24 h recovery period, cells were washed and a final volume of 630 μl buffer-free Assay Medium (Seahorse Bioscience, supplemented with 5 mM glucose (Sigma) and 1 mM sodium pyruvate (Gibco)) was added to each well prior to the experimental protocol. Cells were then kept in a CO_2_-free incubator at 37 °C for 1 h. After instrument calibration, cells were transferred to the XF24 Flux Analyzer to record cellular oxygen consumption rates. Measurements were performed with repetitive cycles of 2 min mixture, 2 min wait, and 4 min OCR measurement times. Injected compounds for the mitochondrial stress test were oligomycin (2 μM working concentration) to inhibit ATP synthase, followed by FCCP (1 μM working concentration) to induce mitochondrial uncoupling and rotenone/antimycin A (2 μM working concentration each) to block the mitochondrial respiratory chain. Injection compounds for the glycolysis stress test were glucose (5.5 mM working concentration), followed by oligomycin (oligo, 2 μM working concentration) to induce maximal glycolytic capacity, and 2-DG (100 mM working concentration) to inhibit glycolysis. Insulin-dependent glycolysis was assessed using a modified glycolysis stress test [70] in which oligomycin was omitted and replaced by Insulin (Ins, 100 nM). Following the assay, the medium was carefully aspirated and cellular protein content was measured using the Pierce BCA Protein Assay Kit (Thermo Scientific) to adjust for potential differences in cell numbers, where indicated.

### Statistical analysis

The significance of differences between means was assessed by two-tailed Student’s t test or analysis of variance (ANOVA) with Sidak post hoc test to adjust for multiple comparisons. Logarithmic transformations were made if the equal variance and normality assumptions were rejected. All measurements were adjusted for confounding effects as indicated. Error bars are expressed as the mean ± SEM unless otherwise specified. p < 0.05 was considered significant.

### Accession numbers

Datasets have been deposited in the Gene expression omnibus archive as SuperSeries GSE139163 combining individual experiments GSE139155, GSE139162, and GSE155781. A reviewer accessible link has been created and is available at http://www.ncbi.nlm.nih.gov/geo/query/acc.cgi?token=ejgdqouubpirhsv&acc=GSE139163 The following secure token has been created to allow review of record GSE139163 while it remains in private status: exyzawkojlsflgr.

## Supplementary Information

Figure S1 (PNG 232 kb)

High Resolution Image (TIF 1048 kb)

Figure S2 (PNG 645 kb)

High Resolution Image (TIF 3269 kb)

Figure S3 (PNG 321 kb)

High Resolution Image (TIF 1789 kb)

ESM 1 (XLSX 383 kb)

ESM 2 (DOCX 39 kb)

## Data Availability

Datasets have been deposited in the Gene expression omnibus archive https://www.ncbi.nlm.nih.gov/geo/ as SuperSeries GSE139163 combining individual experiments GSE139155, GSE139162, and GSE155781.
