# Supplementary figures and images for: LMO3 reprograms visceral adipocyte metabolism during obesity
Source: J Mol Med (Berl). 2021 May 20;99(8):1151–71. doi: 10.1007/s00109-021-02089-9 (PMC8313462; doi:10.1007/s00109-021-02089-9)

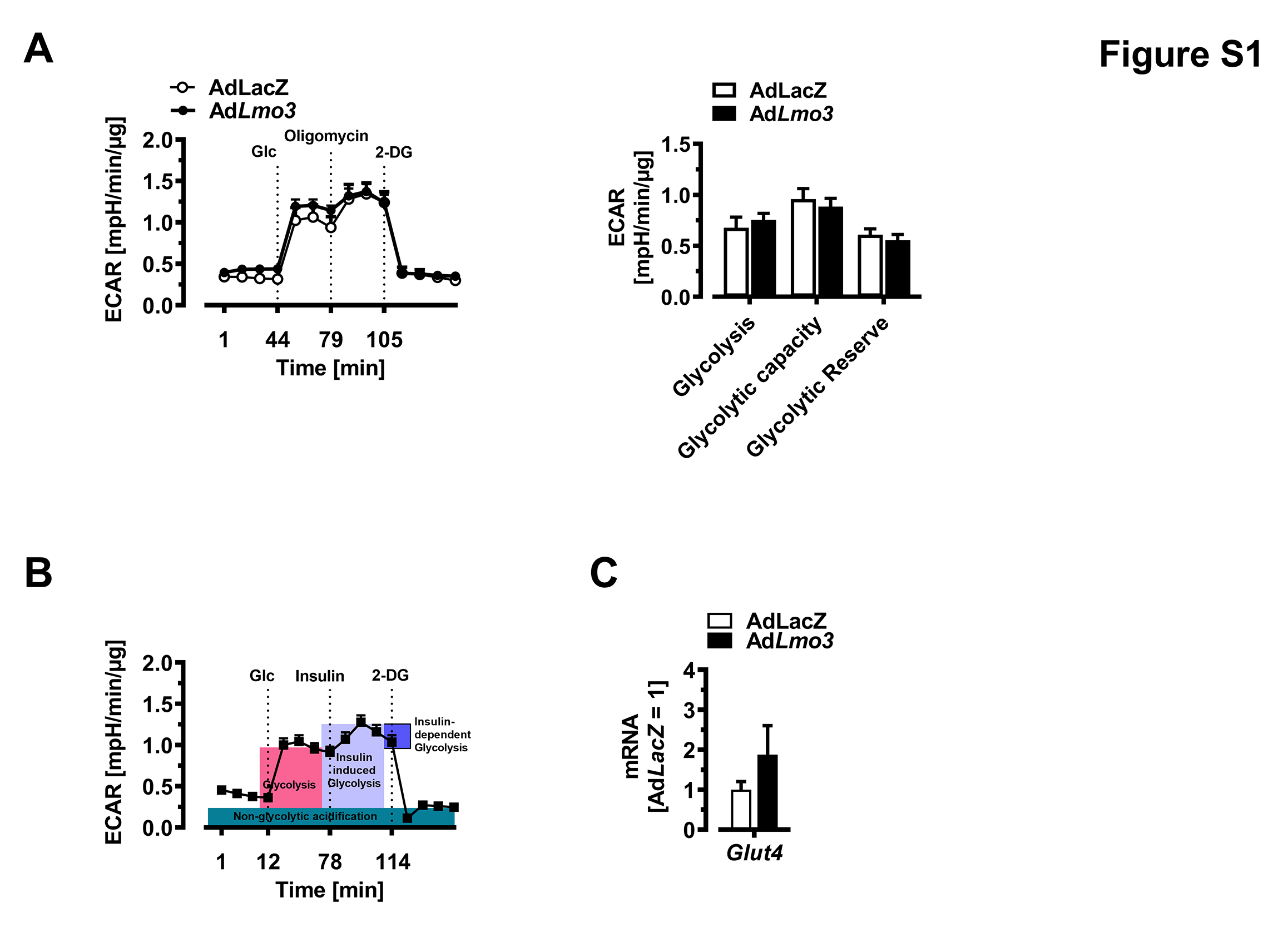

Supplement: Supplementary file 1 — (PNG 232 kb) [file 109_2021_2089_Fig8_ESM.png]

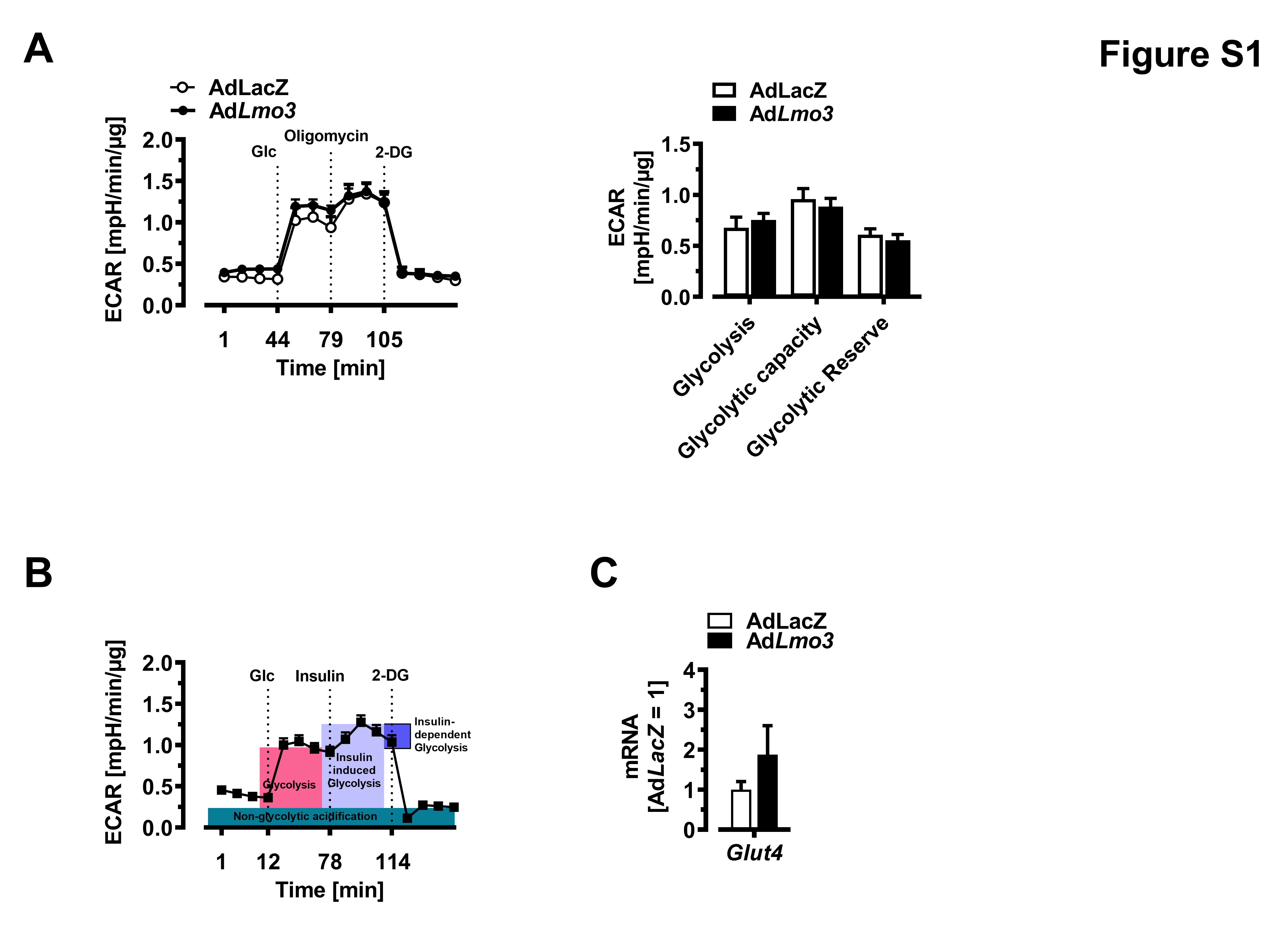

Supplement: Supplementary file 2 — High Resolution Image (TIF 1048 kb) [file 109_2021_2089_MOESM1_ESM.tif]

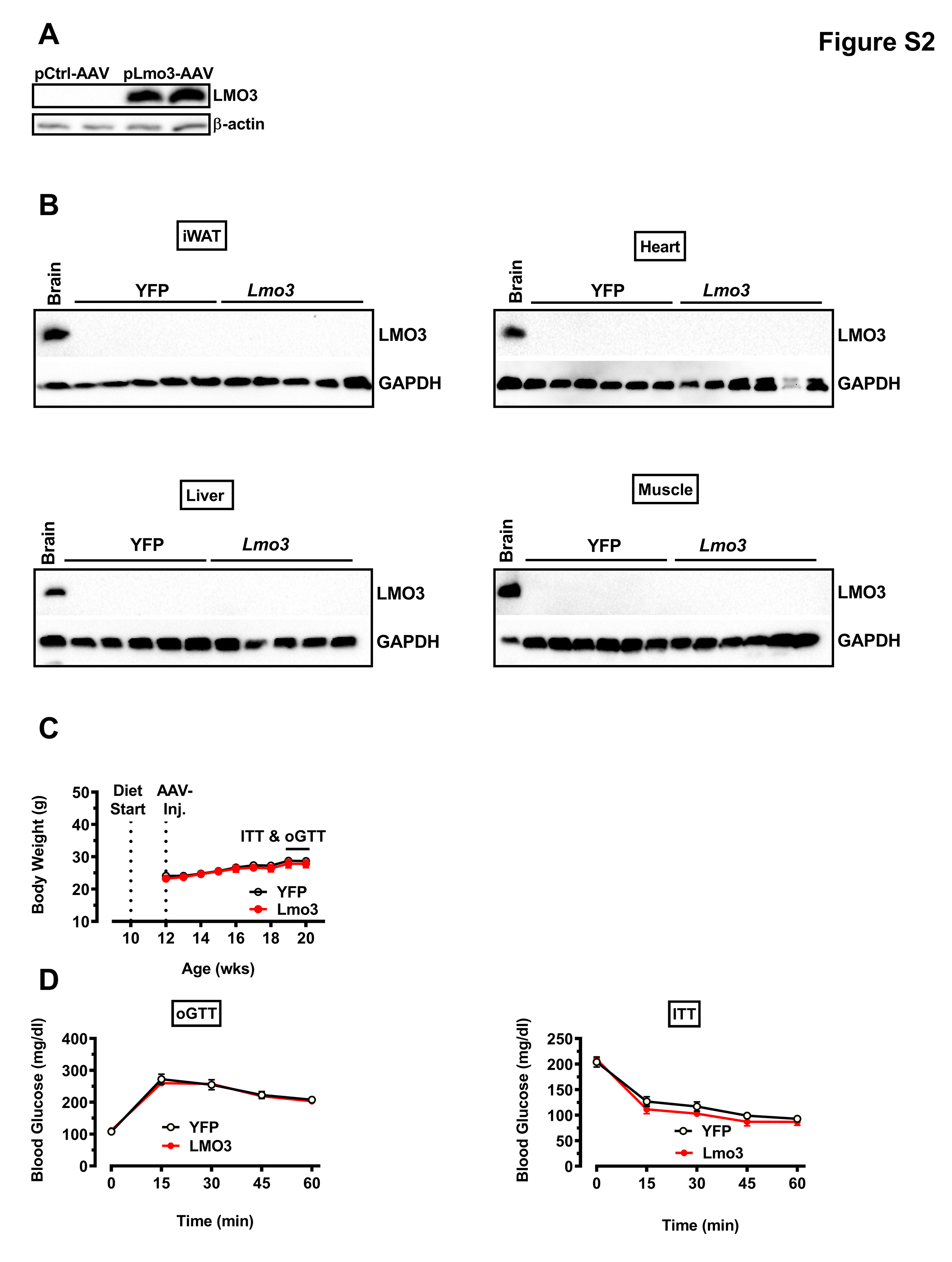

Supplement: Supplementary file 3 — (PNG 645 kb) [file 109_2021_2089_Fig9_ESM.png]

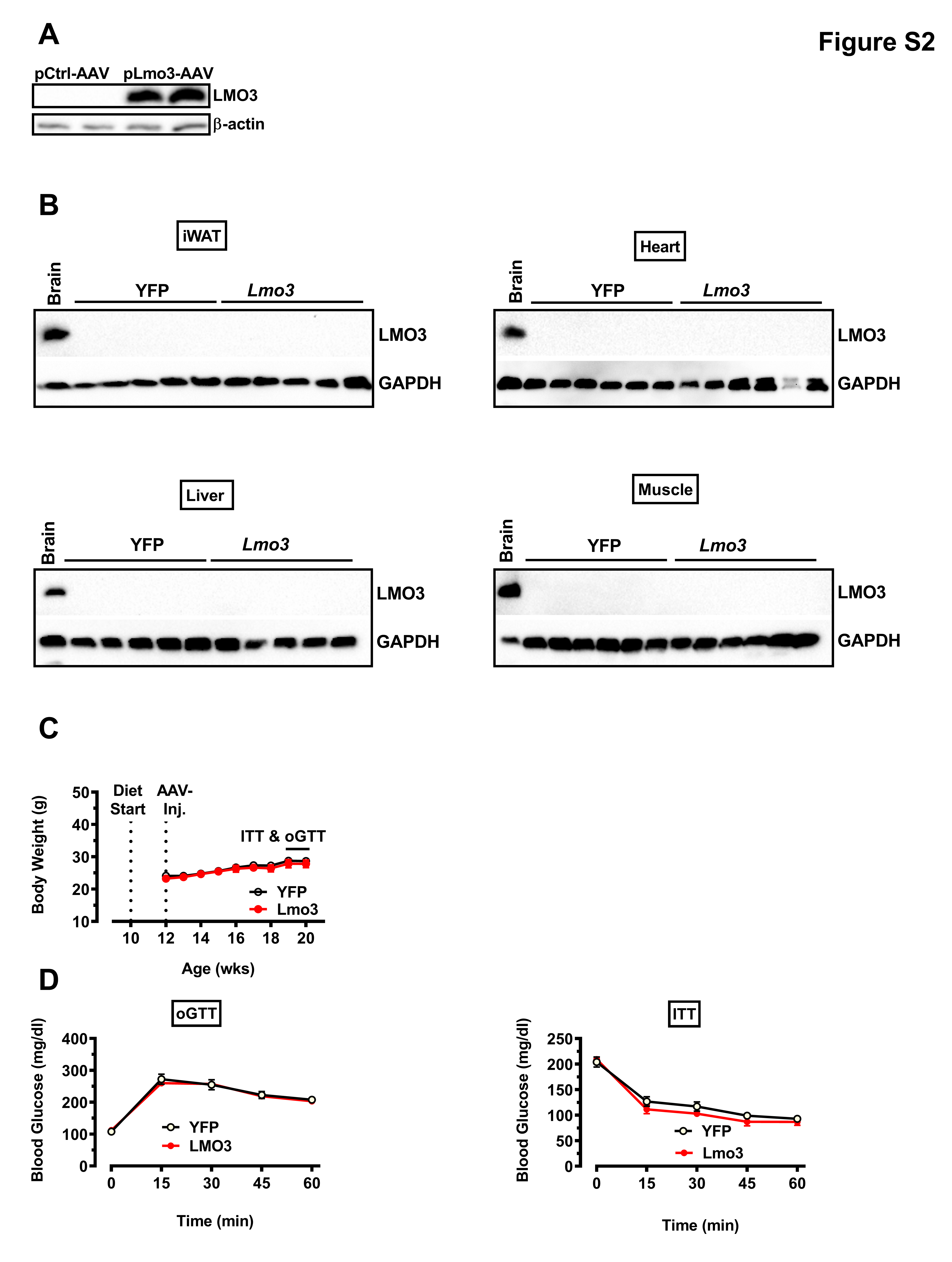

Supplement: Supplementary file 4 — High Resolution Image (TIF 3269 kb) [file 109_2021_2089_MOESM2_ESM.tif]

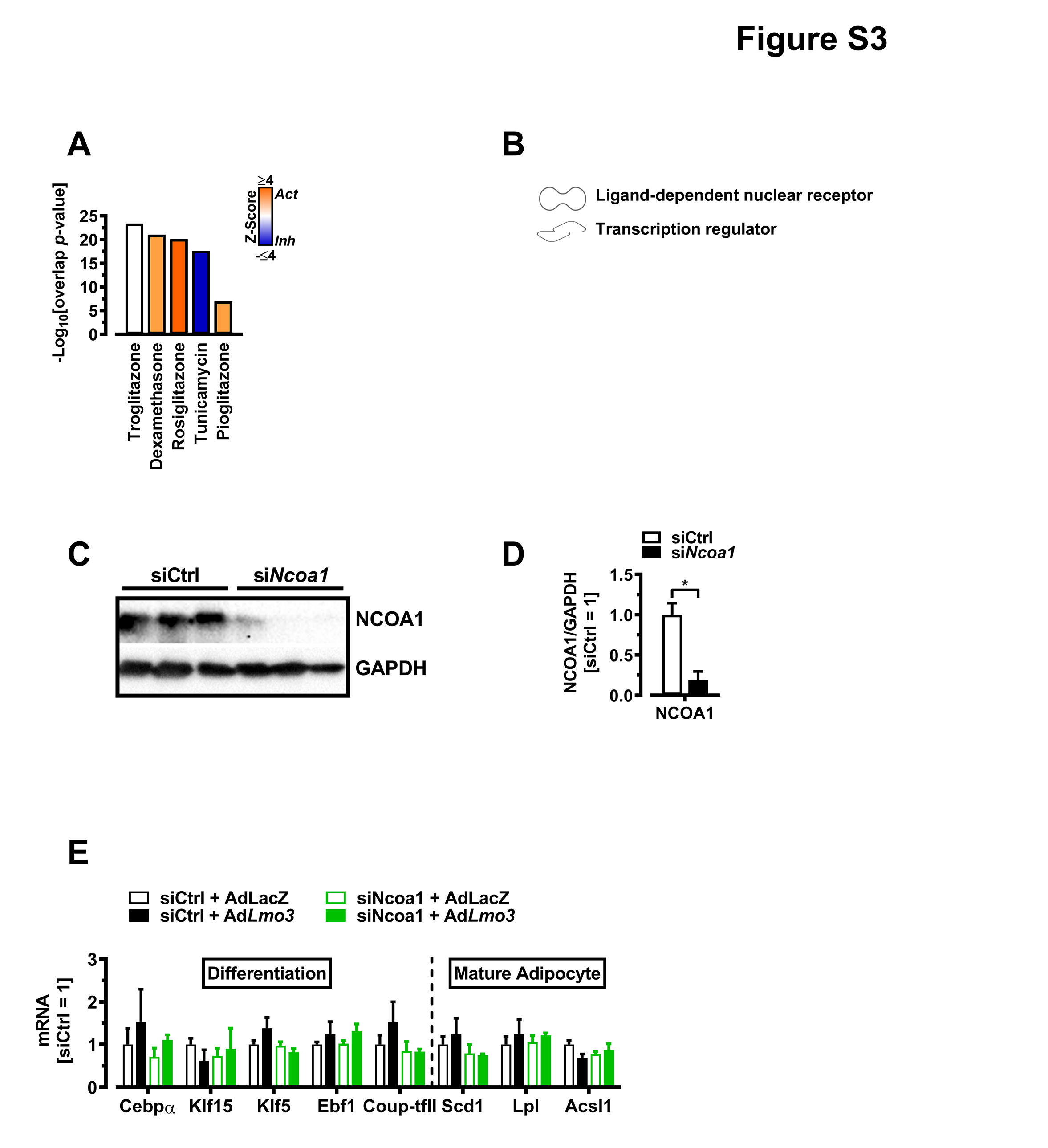

Supplement: Supplementary file 5 — (PNG 321 kb) [file 109_2021_2089_Fig10_ESM.png]

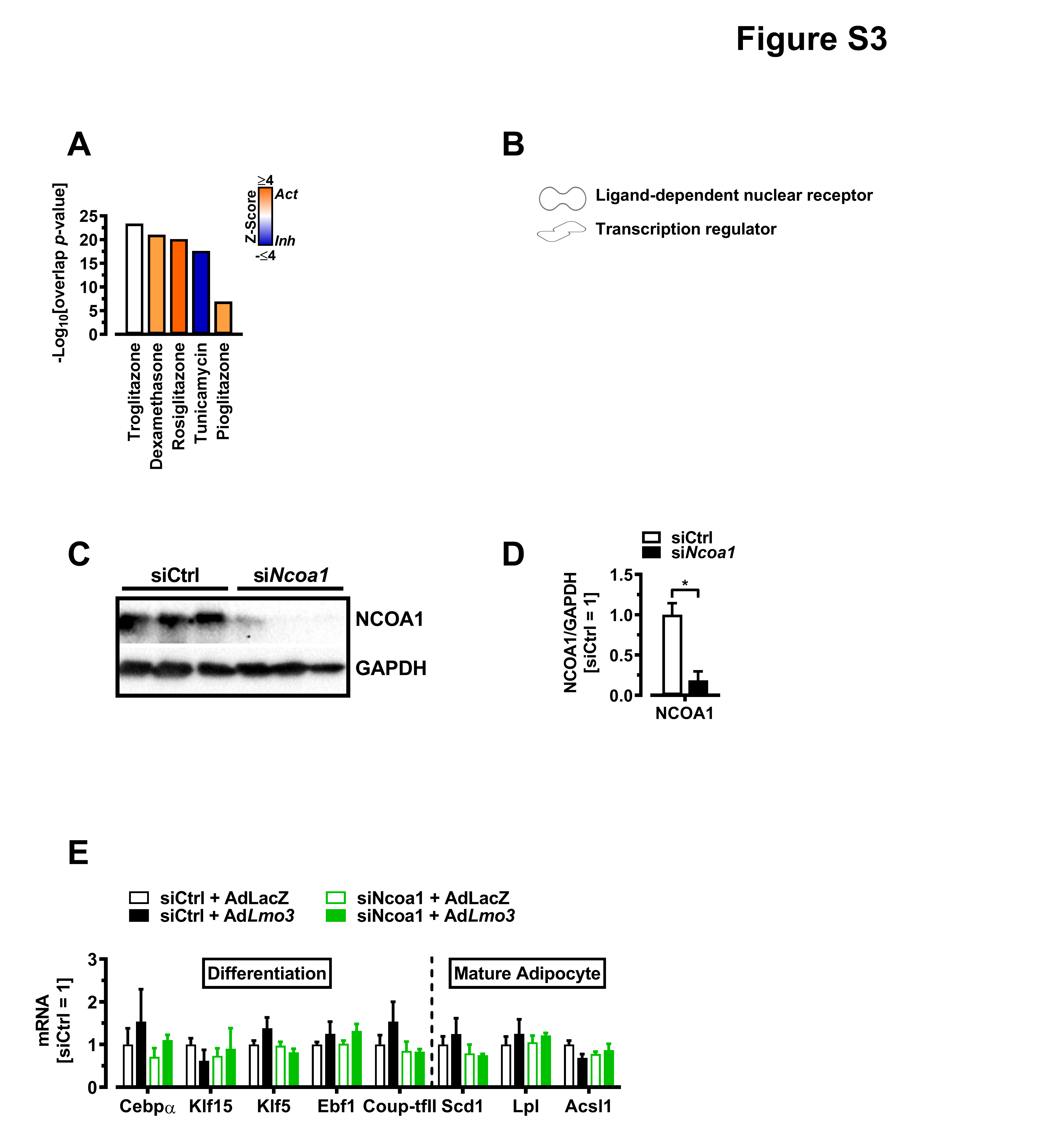

Supplement: Supplementary file 6 — High Resolution Image (TIF 1789 kb) [file 109_2021_2089_MOESM3_ESM.tif]
