# Supplementary material for: LMO3 reprograms visceral adipocyte metabolism during obesity
Source: J Mol Med (Berl). 2021 May 20;99(8):1151–71. doi: 10.1007/s00109-021-02089-9 (PMC8313462; doi:10.1007/s00109-021-02089-9)
Supplement: Supplementary file 8 — (DOCX 39 kb) [file 109_2021_2089_MOESM5_ESM.docx]

**Supplementary information**

**Supplementary Figures**

**Figure S1, *related to Figure 2. LMO3 augments insulin-induced glucose uptake and GLUT4 translocation*.**

**(A)** Glycolysis stress test in AdLacZ or Ad*Lmo3* transduced mature 3T3-L1 adipocytes. ECAR at baseline and after sequential treatment at the indicated time points with glucose (Glc, 5.5 mM), oligomycin (oligo, 100nM) and 2-Deoxyglucose (2-DG, 100mM) in AdLacZ or Ad*Lmo3* transduced mature 3T3-L1 adipocytes. 2-DG was injected to inhibit glycolysis.

**(B)** Schematic representation for determination of Insulin-dependent glycolysis in mature 3T3-L1 adipocytes. Note that Insulin was injected instead of oligomycin, which is used for calculation of maximal glycolytic capacity. The exemplary graph depicts ECAR traces of Ad*Lmo3*-transduced mature 3T3-L1 adipocytes from Figure 2D.

**(C)** *Glut4* mRNA expression in mature 3T3-L1 adipocytes 3 days after infection with a control (AdLacZ) or *Lmo3* containing (Ad*Lmo3*) adenovirus (n = 5-6).

**Figure S2, related to Figure 3. Experimental strategy for an eWAT-specific *Lmo3* knock-in mouse & effects of *Lmo3* expression in eWAT on body weight and whole body metabolism**

**(A)** Western blotting for LMO3 in 293FT cell lysates transfected with AAV plasmids expressing GFP or *Lmo3*.

**(B)** Western blotting for LMO3 in iWAT, liver, muscle and heart. Brain extracts were included as postive control for endogenous LMO3 expression. GAPDH demonstrates equal protein loading. Each lane represents extracted protein from 1 animal. All mice were kept on HFD for 12 weeks and received rAAV-YFP or rAAV-*Lmo3* injections into eWAT at week 2 of HFD and were examined 10 weeks later.

**(C & D)** All mice were kept on a chow diet for 12 weeks and received rAAV-YFP or rAAV-*Lmo3*- injections into eWAT and were examined 10 weeks later. **(D)** Body weight gain over time (n = 5-7). **(E)** Oral glucose tolerance test, insulin tolerance test and corresponding blood glucose in mice fed a chow diet (n = 5-7).

**Figure S3, *related to Figure 6. LMO3 increases mitochondrial oxidative gene expression and glucose uptake via NCOA1/SRC-1.***

**(A)** Ingenuity Pathway Analysis (IPA)-predicted drugs & chemical upstream regulators from Ad*Lmo3-* versus AdLacZ transduced 3T3-L1 adipocytes showing activation Z-score (bars). Drugs & chemicals with an overlap p < 0.05 by IPA were predicted to be upstream regulators.

**(B)** Explanation of molecule shapes used for the IPA-predicted mechanistic network that corresponding to specific transcriptional regulator classes

**(C)** Western blotting of NCOA1 in siCtrl- and si*Ncoa1* treated mature 3T3-L1 adipocytes. GAPDH demonstrates equal protein loading.

**(D)** Densitometry of NCOA1 to GAPDH ratio as shown in (C), (n=3).

**(E)** Q-PCR analysis of genes regulating adipocyte differentiation or mature adipocyte function in siCtr- or si*Ncoa1* transfected control (AdLacZ-) or LMO3-overexpressing (Ad*Lmo3*) 3T3-L1 adipocytes (n=5).

**Supplementary Table S8.** Sequences of oligonucleotide primers.

| Gene symbol | 5' Oligonucleotide | 3' Oligonucleotide |
| --- | --- | --- |
| *ACACA* | TGATCTGGTCACCAAGGAAGC | TCCATGGCTTCCAGGAGTAGC |
| *Acadl* | AGCTGATCGCAAGACAGATCG | AGAATCCGCATTAGCTGCATG |
| *Acly* | GCTATGCCCCAAGGAAAGAGT | CTCGTCTCGGGAACACACG |
| *Aco1* | GGGGTGTGGGTGGTATTGAA | CTTGTCGGAGGTGCTTGGTAAT |
| *Acsl1* | CGCCACCATCAAGATCTAACG | GAAGGCCATTGGTACGAGGAG |
| *Aco2* | GCTGACCCCTCCGACTATAACA | ATGACGCACTTCAGAGGCTTTC |
| *ACOX1* | AACTCCGTGCAGCCAGATTAG | GGTTCCAAGCTACCTCCTTGC |
| *ATGL* | GTGTCAGACGGCGAGAATG | TGGAGGGAGGGAGGGATG |
| *CD36* | GGGAAAGTCACTGCGACATGAT | ACGTCGGATTCAAATACAGCATAGA |
| *Cd36* | GGAGCAACTGGTGGATGGTT | TTGAGACTCTGAAAGGATCAGCA |
| *Cebpα* | GAACAGCTGAGCCGTGAACT | AAACCATCCTCTGGGTCTCC |
| *Coup-TFII* | GCATGAGACGGGAAGCTGTAC | CGTTGGTCAGGGCAAACTG |
| *Cpt1a* | CAAGCCAGACGAAGAACATCG | CCTTCAGCGAGTAGCGCATAG |
| *Cs* | AGCCAAGAACTCATCCTGCC | TCTTCCCATGTTGCTGCTTGA |
| *Dlat* | TGTTCCTCCCACTCCCCAG | CCTTCTGGTCCCGTCCCTTT |
| *Dld* | CAGGTGCTGGAGAAATGGTGA | GCCTCTGATAAGGTCGGATGC |
| *Ebf1* | AGGTTGGATTCTGCTACGAAAGTT | TGATTCCTCTTAAAAAGGCCTGA |
| *Ehhadh* | AGAGGCGCAGGATACCTTGAG | CAGTTGGACTGATGGCATTGAC |
| *Fasn* | TGAACTCCTTGGCGGAAGAGA | GTAGGACCCCGTGGAATGTCA |
| *Hadhb* | GCTGGCTTCTCTGATAAGACTCC | CCAACAGCTGTGGTCATGG |
| *Idh1* | ACTCAGTCGCCCAAGGTTATG | CCCTTTCTGGTACATGCGGT |
| *Idh3a* | CGAGAGAACACGGAAGGAGAA | CGAAGGCAAACTCTGCAATG |
| *Idh3b* | TCACTCCAGCATGATTGCAGAT | GGTGCTGTAGCCTCCCATGT |
| *Klf5* | CCAGACGGCAGTAATGGACAC | TTGACGTCTGTGGAACAGCAG |
| *Klf15* | ACAGGCGAGAAGCCCTTT | CGGTGCCTTGACAACTCAT |
| *LIPE* | CTCAGTGTGCTCTCCAAGTG | CACCCAGGCGGAAGTCTC |
| *LMO3* | AAGGTTGTGCTGGCTGCAAC | GGCACACTTCAGGCAGTCTTC |
| *Lmo3* | GCATGAGGACTGCCTGAAGTG | GCCTTCGTGTACAAGGTGGAG |
| *Mcad* | ATGACGGAGCAGCCAATGA | ATGGCCGCCACATCAGA |
| *Mdh2* | CCGCCTGACCCTCTACGATA | CATCCCGTGTCATTCCTGGTT |
| *Mt-Nd2* | AGGGATCCCACTGCACATAG | CTCCTCATGCCCCTATGAAA |
| *Ncoa1* | GGGAGTGCCAGCATAAAACTG | GGTAGCGTAGGAGCTGATGGT |
| *Ndufv1* | CTTCCCCACTGGCCTCAAG | CCAAAACCCAGTGATCCAGC |
| *Pdha1* | GCTGGCATAAACCCTACGGA | CCTCCTCTTCGTCCTGTTAGC |
| *PLIN1* | GACAACGTGGTGGACACAGT | CTGGTGGGTTGTCGATGTC |
| *PPARG* | AAGGCCATTTTCTCAAACGA | AGGAGTGGGAGTGGTCTTCC |
| *RPLP0* | GTCATCCAGCAGGTGTTCGAC | CTCCAGGAAGCGAGAATGCAG |
| *Rplp0* | GCCAATAAGGTGCCAGCTGCTG | GAAGGAGGTCTTCTCGGGTCCTAG |
| *SCD* | AGCAGGAGCTCATCGTCTGTG | TCATTCTGGAATGCCATTGTG |
| *Sdha* | AAACAGACCTGCGGCTTTCA | AGCATTGATACCTCCCTGTGC |
| *Sdhb* | GCGGACCTATGGTGTTGGAT | GAGCCACAGATGCCTTCTCT |
| *Sdhd* | AGCCCTCACCCTGCACAGT | TCAAAGCTGAGAGTGCCAAGAG |
| *siNcoa1(sense)* | CAGAUUGCCUGAACUAGAAtt |  |
| *siNcoa1(anti-sense)* | UUCUAGUUCAGGCAAUCUGct |  |
|  |  |  |
|  |  |  |

Aliases of gene symbols given in parenthese; capital letters corresponds to human genes.
